# siRNA Machinery in Whitefly (*Bemisia tabaci*)

**DOI:** 10.1371/journal.pone.0083692

**Published:** 2013-12-31

**Authors:** Santosh Kumar Upadhyay, Sameer Dixit, Shailesh Sharma, Harpal Singh, Jitesh Kumar, Praveen C. Verma, K. Chandrashekar

**Affiliations:** 1 National Agri-Food Biotechnology Institute, (Department of Biotechnology, Government of India), Mohali, Punjab, India; 2 CSIR-National Botanical Research Institute, Council of Scientific and Industrial Research, RanaPratapMarg, Lucknow, India; 3 Indian Agricultural Research Institute-Regional Station, Agricultural College Estate, Shivaji Nagar, Pune, Maharashtra, India; University of Kentucky, United States of America

## Abstract

**Background:**

RNA interference has been emerged as an utmost tool for the control of sap sucking insect pests. Systemic response is necessary to control them in field condition. Whitefly is observed to be more prone to siRNA in recent studies, however the siRNA machinery and mechanism is not well established.

**Methodology/Principal Findings:**

To identify the core siRNA machinery, we curated transcriptome data of whitefly from NCBI database. Partial mRNA sequences encoding Dicer2, R2D2, Argonaute2 and Sid1 were identified by tblastn search of homologous sequences from *Aphis glycines* and *Tribolium castaneum*. Complete encoding sequences were obtained by RACE, protein sequences derived by Expasy translate tool and confirmed by blastp analysis. Conserved domain search and Prosite-Scan showed similar domain architecture as reported in homologs from related insects. We found helicase, PAZ, RNaseIIIa, RNaseIIIb and double-stranded RNA-binding fold (DSRBF) in Dicer2; DsRBD in R2D2; and PAZ and PIWI domains in Argonaute2. Eleven transmembrane domains were detected in Sid1. Sequence homology and phylogenetic analysis revealed that RNAi machinery of whitefly is close to Aphids. Real-time PCR analysis showed similar expression of these genes in different developmental stages as reported in *A. glycines* and *T. castaneum*. Further, the expression level of above genes was quite similar to the housekeeping gene actin.

**Conclusions/Significance:**

Availability of core siRNA machinery including the Sid1 and their universal expression in reasonable quantity indicated significant response of whitefly towards siRNA. Present report opens the way for controlling whitefly, one of the most destructive crop insect pest.

## Introduction

Transgenic crops expressing δ-endotoxins of *Bacillus thuringienesis* (Bt) provide incredible control of chewing type lepidopteran and coleopteran pests [1]. However, they are completely failed against sap sucking hemipteran insects like aphids, mealybugs, whiteflies and others [2], [3]. These sucking pests are now emerged as major pests in crop field. Some of the plant lectins are reported to be effective against these insects, but none of them are toxic to whiteflies. RNA interference (RNAi) has been reported as good alternative to combat these issues. Although most of the RNAi studies are focused on regulation, expression of target genes and mechanism of small RNA in the insects [4], [5]; yet the development of insect resistant transgenic plants expressing dsRNA/siRNA is becoming more popular due to their target specificity [6]–[9].

RNAi can be triggered by both exogenous and endogenous dsRNA/siRNA, which silences the endogenous target gene having similar sequence. RNAi has been described in various insect orders including hemiptera [10]. siRNA and miRNA pathways are reported as two overlapping pathways for RNA mediated gene silencing. Both siRNA and miRNA pathways use related but discrete protein molecules at each step of their activity. Dicer1, Loquacious and Argonaute1 are involved in miRNA pathway in *Drosophila*, while Dicer 2, R2D2 and Argonaute2 function in siRNA mediated pathway [11]–[15]. *Tribolium castaneum* is reported as model organism among insects for systemic silencing by RNAi [16]–[18] because *Drosophila melanogaster* do not show systemic RNAi response due to the absence of gene called systemic RNA interference deficient-1 (Sid1) [19], [20]. Sid1 is accountable for scattering the intensified signal for RNAi [21]. A few reports are available for Sid1 in insects and further identification and characterization of homologous sequences is in progress from other insects [18], [22], [23]. siRNA mediated control of insects like whitefly can be accelerated by understanding the pathway and mechanism of systemic silencing. High level expression of Sid1 in target insects might act as an indicator for systemic RNAi response.

Whitefly (*B. tabaci*) is reported as a serious pest of several crops. Further, none of the reported insecticidal proteins are significantly effective against them. However, in our previous study, we have shown RNAi as a good tool for the control of whitefly [24]. Although we found that some of critical gene targeted by siRNA molecules (like vATPaseA, RPL 9) are very effective [24], but the RNAi machinery and their mechanism in whiteflies are still unknown. Recently, availability of transcriptome data of whitefly on NCBI [25], [26], [27] opens the possibility of exploring RNAi machinery. Present study aimed to identify and characterize major components of siRNA machinery like Dicer2, R2D2, Argonaute2 and Sid1 in whiteflies. We found the presence of complete siRNA machinery in whitefly and significant expression level in different developmental stages.

## Materials and Methods

### Mining of cDNA encoding Dicer2, R2D2, Argonaute2 and Sid1 from transcriptome data

To identify the Dicer2, R2D2, Argonaute2 and Sid1 encoding cDNA from whitefly transcriptome data, homologous proteins sequences (AFZ74931, AFZ74932, AFZ74933 and AFZ74934 for Dicer2, R2D2, Argonaute2 and Sid1 of *Aphis glycines*; and NP_001107840.1, NP_001128425.1, NP_001107842.1 and NP_001099012.1 for Dicer2, R2D2, Argonaute2 and Sid1 of *T. castaneum*, respectively) were downloaded from NCBI database [28]. These sequences were used for tblastn (http://blast.ncbi.nlm.nih.gov/Blast.cgi) search against transcriptome data (i.e. TSA) of *B. tabaci* and matching TSA sequences were retrieved. To further confirm the identity, TSA sequences were analysed using blastx search against NCBI non-redundant proteins database.

### Cloning of full length genes by RACE

Sequences identified in above blast analysis were used for primer designing to obtain complete gene sequence by RACE. Sequences used were as follows- EZ956195.1 for R2D2, HP663253.1 for Argonaute 2 and EZ964892.1 for Sid1. In case of Dicer2, we designed primers from two TSA sequences (EZ956963.1 and EZ954838.1) to ease the amplification, cloning and sequencing, because of its large size. Primers used for RACE are given in Table 1. Both 5′ and 3′ RACE were performed using the RACE Kit (Clontech, USA) following the standard protocol provided by manufacturer. Amplified cDNA were cloned in TA cloning vector and sequenced using ABI3730 XL DNA analyser (Applied Biosystems, USA). Both 5′ and 3′ RACE sequences were assembled together to obtain the complete genes sequences.

**Table 1:** Primer used in RACE of siRNA components of whitefly.

		5′ RACE (5′-3′)	3′ RACE (5′-3′)
Dicer2	GSP1	GTTGATCGAACTGCTGATTCCAC	GGAAGGAGCAGCCTCAGGGTT
	NGSP1	CTTACAGCTTGCGCCTGTTGCTC	CGGGAGTACCAAACATTGATCATG
	GSP2	TTTGCCCTCATCAATTCCTCCGC	GCACTTGGAGACCCTCCTCAG
	NGSP2	CTCCAAGTGTTTCCAAGTACTCG	GGACCCCAACAGTGCGAGATC
R2d2	GSP	CTTGAAGGTTCTTCAACCTTGTCAAC	GAGCCTACTGAGGCCTTACCCA
	NGSP	CTGACAGTAAACAATTTAGCATGGG	ATTCTGTTGGTGCATTAACTGAGTTC
Argonaute 2	GSP	TTCTGGTCCATTGTCCGGACGG	GAACTCAAGAACGACTGGGCAGC
	NGSP	TGTCCCTTGGATTGTTCAATCTGC	GCTGCGCCGTTAGTTTCTGGTC
Sid 1	GSP	GTATATTATTGCCATGTTATCCATGC	GTCCTCTTGAAAGAATGAAATCCTAG
	NGSP	ACTTATCTAGTCCTTGCTTGTGTG	GTGGAGTGCAGTGAAAACAACGTAG

### Sequence analysis

To get the open reading frame, each gene sequence was subjected to ORF finder (http://www.ncbi.nlm.nih.gov/gorf/orfig.cgi) at NCBI database. ORFs of all the genes were further confirmed by blast against NCBI database. Encoding protein sequences were derived by Expasy translate tool (http://web.expasy.org/translate/). Theoretical molecular mass and pI of translated sequences were determined by Expasy MW/pI tool (http://web.expasy.org/compute_pi/).

To analyse the domain architecture of whitefly Dicer2, R2D2, Argonaute2 and Sid1; derived protein sequences were subjected to Scan-Prosite (http://prosite.expasy.org/scanprosite/), a database of protein families and domains [29]. It contains pattern and profile specific for thousands of protein families or domains. Sid1 sequence was analysed by TMHMM server v2.0 (http://www.cbs.dtu.dk/services/TMHMM/) and InterProScan (http://www.ebi.ac.uk/Tools/pfa/iprscan/) to detect the transmembrane helices. TMHMM is a server which predicts transmembrane protein topology with hidden Markov model. Gene sequences encoding *B. tabaci* Dicer2, R2D2, Argonaute2 and Sid1 were submitted to NCBI database (Table 2).

**Table 2:** Details of whitefly *Dicer2, R2D2, Argonaute2 and Sid1 sequences*.

Gene	Accession Number	Orf length	Protein length (AA)	Molecular mass (kDa)	pI	Closest homolog	% Similarity
Dicer2	KF740508	4944	1647	189.0	5.91	*Blattella germanica* CCF23094.1	38
R2D2	KF740509	759	252	27.7	6.98	*Acromyrmex echinatior* EGI67607.1	40
Argonaute2	KF192313	2505	834	95.1	9.48	*Nilaparvata lugens* AGH30327.1	51
Sid1	KF192314	2181	726	83.1	5.79	*Locusta migratoria* AFQ 00936.1	51

### Multiple sequence alignments (MSA) and phylogenetic analysis

Multiple sequence alignments were performed with the well-known insect sequences to analyse the homology, and presence of conserved domains and amino acids sequences. Sequences used for alignment are given in table S1. Phylogenetic analyses were performed by MEGA 5.2.1 software. Conserved domains used in phylogenetic analysis were –RNaseIIIa and b of Dicer2 and DsRBD of R2D2. Further full length protein sequences of Dicer2, R2D2, Argonaute2 and Sid1 were also used in phylogenetic analysis. Sequence alignments were performed using Muscle. Neighbour joining analysis was performed with boots trapping test using 10,000 replicates. Maximum likelihood analysis [30] was also performed for the same alignments; however both the analysis showed similar relationship.

### Expression analysis of core components of siRNA pathway in different developmental stages of whitefly

For experimental purpose, we reared whiteflies in control condition on cotton plants as described earlier [24]. Total RNA was isolated from egg, nymph and adult insects (∼10 mg each) using Tri reagents (Sigma, USA). cDNA was synthesized from 2 µg of total RNA using first strand cDNA synthesis kit (Invitrogen, USA). Quality of cDNA was analysed by PCR amplification of *actin* gene. cDNA from different stages of insects was used for expression analysis of Dicer2, Argonaute2, R2D2 and Sid1 by real time PCR on GeneAmp 5700 (Applied Biosystems, USA) using SYBR Green detection dye (Invitrogen, USA). Primers used for real time PCR is provided in Table 3. Amplification of *actin* gene was used as control. Expression analysis experiment was performed in triplicates.

**Table 3:** Primers used in real time PCR of siRNA components of whitefly.

Gene	Forward primer (5′-3′)	Reverse Primer (5′-3′)
Dicer2	CAGCCTCAGGATTTACTC	CCTGCTCCTGTAGGCAAG
R2D2	GTCCGTGATGATACTGGTAC	GGACGAACCAGTTCCCTC
Argonaute2	GGCCACAGCCTGGACAAT	CCCTGTGACGCAAGCATTCTA
SID1	CACACCTTCAGAGCCAGCATTC	TGTTTTGGATGGATAGGGTCATG
Actin	GACCAGCCAAGTCCAAACGA	CCTTTGTGGTAGAGGTCTCAGTT

## Results and Discussion

### Identification and cloning of core components of siRNA pathway in whitefly

Usually the core components of siRNA machinery are highly conserved within species, however the depth of conservation often differs between the species. Further the efficiency of RNAi and degree of systemic response also varies from species to species. In certain organisms like *C. elegans* and *Tribolium*, injection of a small amount of dsRNA induces significant systemic response [17], [21]. However, some lepidopteran insect do not show such kind of response [31]. Therefore, understanding of molecular machinery of RNAi is pre-requisite in different insects. Presence and absence of the components of RNAi machinery (especially Sid1 protein) in an organism might be an indicator for their response. Therefore, we surveyed for the presence of core components of siRNA machinery in whitefly, a devastating insect pest of several crops.

We mined the transcriptome data of whitefly for the presence of Dicer2, Argonaute2, R2D2 and Sid1 by using homologous sequences from *A. glycines* and *T. castaneum* [28]. In this process, we identified mRNA sequences EZ956963.1 and EZ954838.1 for Dicer2, EZ956195.1 for R2D2, HP663253.1 for Argonaute 2 and EZ964892.1 for Sid1; which were used in primer designing. Gene specific primers (Table 1) were designed and complete gene sequences obtained by RACE (File S1). Sequences were submitted to NCBI, accession numbers, molecular weight, pI and highly homologous protein to each sequence is given in Table 2. Whitefly Dicer2, R2D2, Argonaute2 and Sid1 showed high homology with *Blattella germanica* (accession number CCF23094.1), *Acromyrmex echinatior* (EGI67607.1), *N. lugens* (AGH30327.1) and *Locusta migratoria* (AFQ00936.1) protein sequences, respectively.

### Dicer

**Figure 1:**
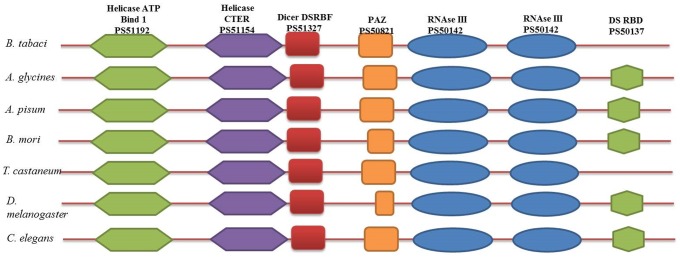
Domain architecture comparison of whitefly Dicer2 protein with other insects and *C. elegans*. Whitefly Dicer2 showed the presence of all the domains except DSRBF as reported in *T. castaneum*.

Dicer is a multi-domain protein basically involve in generation of small RNA molecules (siRNA, miRNA) [32], [33]. A typical Dicer contains two N-terminus helicase domains, one PAZ domain, tandem RNaseIII domains and a c-terminus dsRNA binding domain (Figure 1). In case of *C. elegans*, single Dicer protein is responsible for both miRNA and siRNA pathway [33]–[35]. However, both the pathways are governed by two different Dicers (Dm-Dcr1 and Dm-Dcr2) in *Drosophila* [12]. Dm-Dcr2 is involved in siRNA pathway, whereas Dm-Dcr1 in miRNA pathway. Similar kind of gene duplication is also reported in aphid *Acyrthosiphon pisum* [36].

We retrieved whitefly TSA sequences (accession numbers - EZ956963.1, EZ957381.1, EZ955681.1, EZ954014.1, EZ954838.1, EZ954888.1, EZ939818.1, HP647437.1, EZ942655.1, EZ942655.1, EZ947031.1) showing significant similarity in tblastn search with *A. glycines* and *T. castaneum* Dicer2 protein sequences. Blastx search of retrieved sequences at NCBI-nr protein database indicated the presence of two Dicer (Dicer1 and Dicer2) proteins in whiteflies, as reported in case of other insects [12], [36]. It is possible that Dicer1 is involved in miRNA pathway and Dicer2 in siRNA pathway. Since, we were focussing on characterization of siRNA machinery; we amplified the *dicer2* gene only. Scan-Prosite search of whitefly Dicer2 protein sequence showed the domain organization similar to *T. castaneum* Dicer2 (Figure 1). C-terminus double stranded RNA binding domain (DsRBD, PS50137) was absent in whitefly, as reported in *T. castaneum*. However, other domains like helicase, double-stranded RNA-binding fold (DSRBF), PAZ and RNaseIII were similar to other analysed insects. Whitefly Dicer2 contains two helicase, one DSRBF, one PAZ and two RNaseIII (a and b) domains. Scan-Prosite analysis showed significant score for helicase I (21.6), II (12.8), PAZ (16.1), RNaseIIIa (14.5) and b (34.0) domains (Table 4), which were similar to other insects. However, whitefly and *T. castaneum* lacks the C-terminus DSRBD domain and *D. melanogaster* lacks full-length PAZ domain.

**Table 4:** Scan-Prosite score for common domains of Dicer2 protein in selected insects.

	Dicer2					
Domains	HELICASE 1	HELICASE 2	PAZ	RNAse III (a)	RNAse III (b)	DSRBD
*B. tabaci*	21.6	12.8	16.1	14.5	34.0	Absent
*A. glycines*	19.4	13.9	17.3	20.0	35.5	9.9
*A. pisum*	19.6	13.6	18.4	19.6	33.9	9.6
*B. mori*	21.3	12.6	11.9	21.3	34.6	9.0
*C. elegans*	22.7	14.6	23.5	23.7	40.6	11.9
*D. melanogaster*	18.6	12.1	8.7	18.4	31.0	9.6
*T. castaneum*	22.1	12.0	17.1	23.8	36.8	Absent

**Figure 2:**
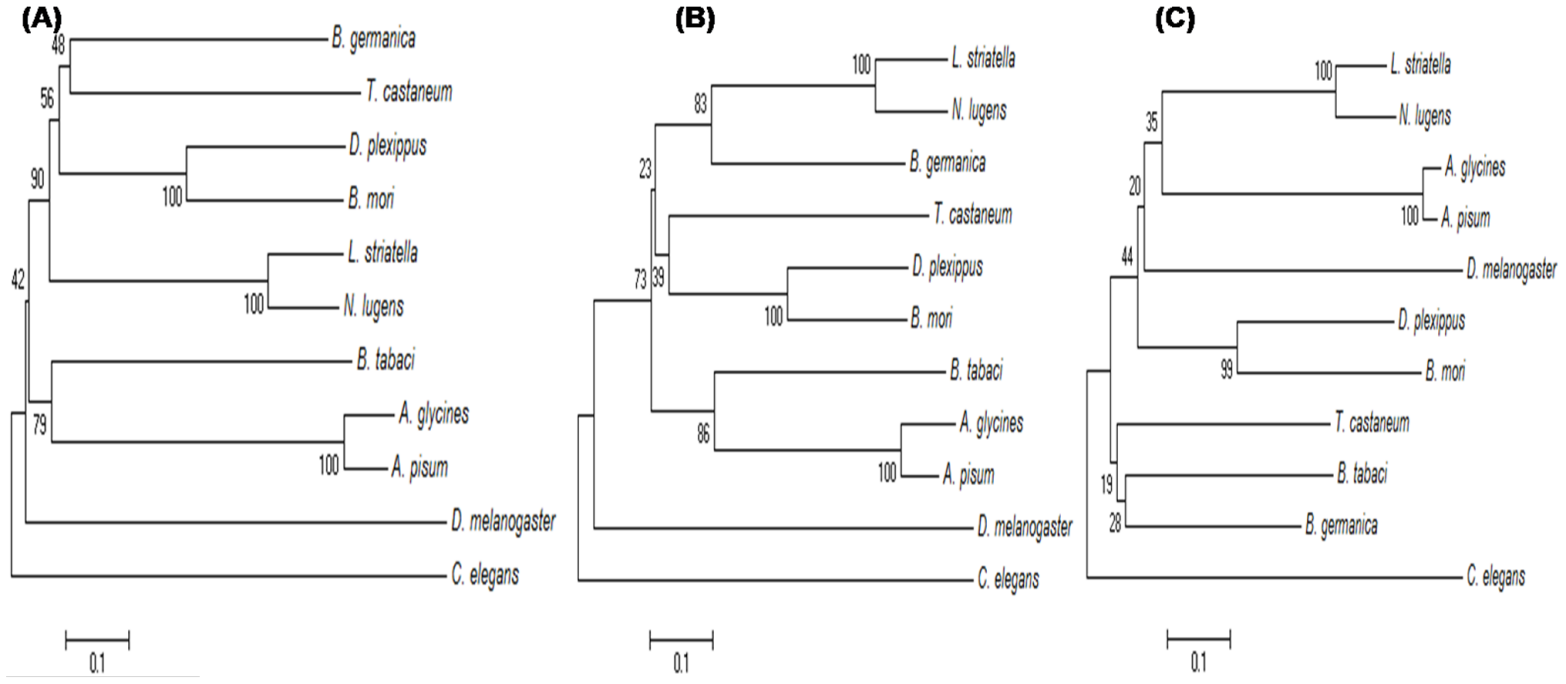
Phylogenetic analysis of whitefly Dicer2 protein with other insects and *C. elegans*. Phylogenetic trees were constructed from amino acid sequences of (**A**) full length protein and (**B**) RNAseIIIa and (**C**) RNAseIIIb domains of whitefly Dicer2 clustered with aphids.

Multiple sequence alignments and phylogenetic analysis of Dicer2 were performed using full length protein as well as RNAseIIIa and b domains sequences from different insects (Table S1, File S2, Figure 2a b and c). Insect Dicer2 proteins were clustered in two groups apart from *D. melanogaster*. Full length whitefly Dicer2 clustered with aphids. However RNaseIIIa and b domains were clustered with aphids and *B. germanica*, respectively. Multiple sequence alignment results also supported the phylogenetic results. Whitefly RNaseIIIa showed ∼45% homology with aphids; however RNaseIIIb showed ∼63% with *B. germanica* (File S2). Results indicated that the two domains might evolve independently during evolution.

### Argonaute

Argonaute is a core component of miRNA and siRNA pathways [37], [38]. It contains two distinctive domains i.e. PAZ and PIWI [38]. Besides these, DUF1785 domain is also reported, however its function is still unknown. PAZ domain is responsible for siRNA binding at 2 nucleotide 3′ overhang, while PIWI domain shows RNaseH like activity. Argonaute is reported as large family of protein in *C. elegans* and *Drosophila* with different functions [11], [39]. Five different Argonautes are reported in *Tribolium* and *Drosophila* [18], in which Argonaute1 and 2 are involved in miRNA and siRNA pathway, respectively [11]. Similar kind of gene diversification is also observed in case of *A. pisum* [36]. However, single Argonaute is reported from *A. glycines* [28].

**Figure 3:**
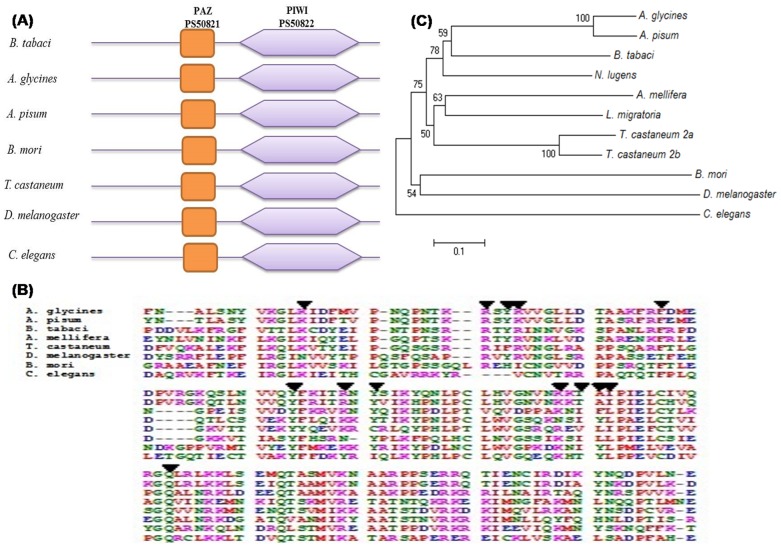
Domain architecture and phylogenetic analysis of whitefly Argonaute2 protein. (**A**) Comparative domain architecture of whitefly Argonaute2 with other insects and *C. elegans*. Both PAZ and PIWI domains are present in all the analysed sequences. (**B**) Alignment of PAZ domain with other insects and *C. elegans* sequences. Figure shows high degree of homology between aligned sequences. Triangles indicate the presence of signature sequences responsible for efficient binding with siRNA [40]. Almost all the signature sequences are present in whitefly. (**C**) Phylogenetic analysis of whitefly Argonaute2 with other insects and *C. elegans*. Tree was constructed on the basis of full length sequence. Figure shows that the whitefly Argonaute2 clustered with *N. lugens* and aphids showing high homology between them. Similar result is observed in multiple sequence alignment (File S3).

Tblastn search of *T. castaneum* and *A. glycines* Argonaute2 sequences against whitefly transcriptome data at NCBI showed similarity with HP663253.1, HP822302.1, HP662784.1, EZ960356.1, and EZ961415.1 TSA sequences. These TSA sequences were retrieved and used for blastx search at NCBI-nr protein database, which indicated the presence of both Argonaute1 (HP663253.1) and 2 (EZ961415.1) in whitefly. However, we cloned the *argonaute2* only because we were interested to explore the siRNA machinery (Table 2). Like other insect's Argonaute2, whitefly Argonaute2 also contains PAZ and PIWI domain (Figure 3a, Table 5). PAZ domain sequence was analysed for the presence of important amino acids actively involved in binding with siRNA [40]. We found almost all these residues, and they were highly conserved among analysed insect's sequences (Figure 3b).

**Table 5:** Scan-Prosite score for common domains of Argonaute2 protein in selected insects.

	Argonaute2	
	PAZ	PIWI
*B. tabaci*	18.2	46.8
*A. glycines*	15.8	44.3
*A. pisum*	16.7	44.9
*B. mori*	11.9	34.8
*C. elegans*	31.1	50.8
*D. melanogaster*	14.1	42.2
*T. castaneum*	12.7	38.2

PIWI domain of Argonaute2 was also found highly conserved (File S3). PIWI domain sequence was analysed for the presence of signature residues involve in binding with siRNA/miRNA. It is reported that three non-bridging oxygen atoms at 5′ phosphate of siRNA involve in interaction with several amino acids of PIWI domain [41]. We observed the presence of these amino acids in whitefly and fount that they were highly conserved in different organisms (File S3).

Phylogenetic analysis of whitefly Argonaute2 was performed with selected insects (Figure 3c). It was grouped with *N. lugens* and aphids. Multiple sequence alignment of PIWI domain showed high homology with *N. lugens* and aphids sequences followed by *L. migratia* and *T. castaneum* (File S3). These results indicate close homology of whitefly Argonaute2 with other related insects.

### R2D2

R2D2 and Loquacious are family of dsRNA-binding proteins and function in tandem with specific RNaseIII enzymes. There are two dsRNA-binding domains in R2D2 and three in Loquacious. Two distinct Dicer complexes, Dcr1/Loquacious and Dcr2/R2D2 are reported in *Drosophila*, which produce miRNA and siRNA, respectively [13], [14], [15], [42]. Loquacious enhances miRNA producing activity of Dcr1 by increasing the affinity toward pre-miRNA, however R2D2 is not directly involve in siRNA producing activity of Dcr2 [13], [14], [42]. Dcr2/R2D2 complex binds to duplex siRNA, forms the RISC loading complex, and enhances siRNA transfer to Argonaute2 [14], [43], [44].

**Figure 4:**
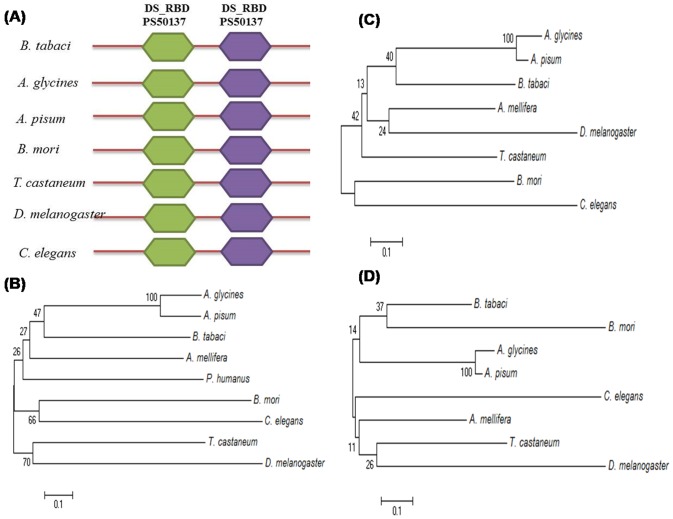
Domain architecture and phylogenetic analysis of whitefly R2D2 protein. (**A**) Comparative domain architecture of whitefly R2D2 with other insects and *C. elegans*. Figure shows two DSRBD in whitefly R2D2 as reported in other insects and *C. elegans*. (**B**) (**C**) and (**D**) Phylogenetic analysis of full length, and DSRBD1 and DSRBD2 domains of whitefly R2D2, respectively. DSRBD1 clustered with aphids; however DSRBD2 clustered with *B. mori*, indicating independent evolution of both the domains.

Tblastn analysis of homologous sequence from *A. glycines* and *T. castaneum* against NCBI TSA database of whitefly showed the presence of both R2D2 (EZ956195.1) and Loquacious (HP798110.1), as reported in other insects. However, we performed the detail characterization of R2D2 only. Similar to other insects, domain architecture analysis at Scan-Prosite showed two double stranded RNA binding domains (DSRBD, PS50137) in whitefly R2D2 (Figure 4a, Table 6). Multiple sequence alignment of R2D2 protein showed significant homology with other insects (File S4). As expected, maximum similarity was observed with aphid *A.* glycines, followed by *P. humanus* and *A. mellifera*.

**Table 6:** Scan-Prosite score for common domains of R2D2 protein in selected insects.

R2D2		
	DSRBD 1	DSRBD 2
*B. tabaci*	14.8	15.4
*A. glycines*	14.4	13.7
*A. pisum*	14.8	13.9
*B. mori*	15.8	15.2
*C. elegans*	10.9	9.5
*D. melanogaster*	12.4	11.7
*T. castaneum*	14.4	14.5

Phylogenetic analyses were performed with full length as well as DSRBD1 and DSRBD2 domains sequences of whitefly R2D2 with other insect sequences. Full length R2D2 and DSRBD1 were clustered with aphids. DSRBD2 was clustered with *B. mori*, however closely followed by aphids (Figure 4b, c and d). Multiple sequence alignment also supports the phylogenetic results. Overall, we found both R2D2 and Loquacious in *B. tabaci*; which might be involved in two parallel siRNA and miRNA pathways, respectively.

### Sid 1

It is the best known protein for systemic RNAi in *C. elegans* [21], [45] and insects [18]. It comprises tandem repeats of transmembrane domains along with long N-terminus extracellular domain. Transmembrane domains form channel for the movement of dsRNA molecules [21], [45]. Sid1 is reported from several insects like *T. castaneum, A. mellifera, A. glycines, B. mori* and others, and involved in systemic spreading of RNAi [18], [28]. However, it is absent in *Drosophila* which lacks the systemic RNAi response. Tomoyasu et al. [18] performed robust analysis of Sid1 gene from several insects genome including 11 *Drosophila* species and tried to correlate the presence and absence of Sid1 gene with RNAi response, however it is still under debate [22], [23], [46], [47], [48]. Over-expression of *C. elegans* Sid1 in *Drosophila* culture cells enables them to uptake dsRNA from media, which confirmed the role of Sid1 in dsRNA uptake [45]. However, Luo et al [23] reported that Sid1 is not required for systemic RNAi in the migratory locust *Locusta migratoria*. This showed that role of insect Sid1 in systemic silencing is still a matter of profound investigation.

**Figure 5:**
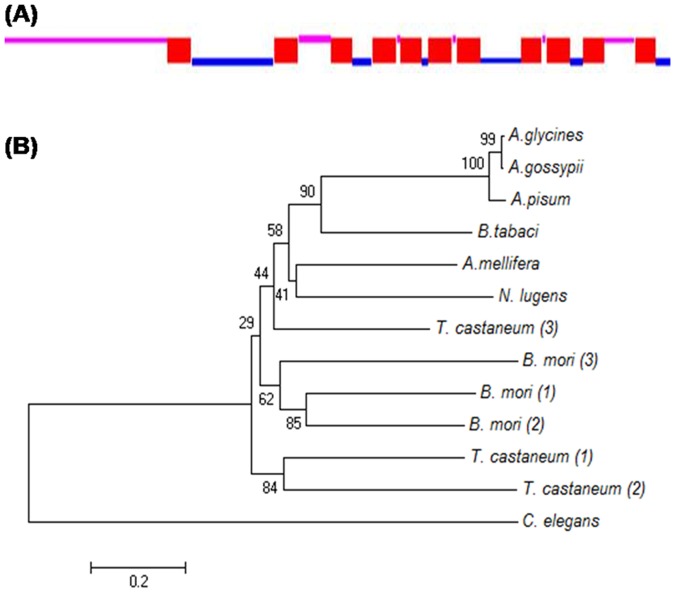
Domain architecture and phylogenetic analysis of whitefly Sid1. (**A**) Domain architecture of whitefly Sid1. Figure shows 11 transmembrane domains (red box) separated by extracellular (pink line) and intracellular (blue line) domains. Long N-terminus extracellular domain is present as reported in other insects. Domain architecture is similar to other insects [28]. (**B**) Phylogenetic analysis of whitefly Sid1 with other insects and *C. elegans*. Figure shows that whitefly Sid1 clustered with aphids, which complements the result of multiple sequence alignment (File S5).

After deep analysis of transcriptome data available at NCBI [25], [26], [27], we found that at least one Sid1 gene is present in whitefly, as observed in case of aphids [28]. Full length gene was obtained by RACE, which encodes for 726 amino acids residue long protein (Table 2, File S1). Blastp analysis at NCBI-nr protein database confirmed that the cloned gene was Sid1. Domain architecture of Sid 1 was analysed by TMHMM server version 2.0 and InterProScan, which showed the presence of 11 transmembrane domains separated by extra and intracellular domains (Figure 5a). Besides this, a long extracellular domain was located at N-terminus. Similar kind of domain organization has been reported from aphids also [28]. The extracellular domain contains three conserved regions (File S5) earlier reported in several organisms. Along with insects, region 1 and 3 are also reported to be conserved in nematodes and vertebrates [18]. Multiple sequence alignment of Sid1 with several insects showed high degree of homology, especially in transmembranes regions and extracellular conserved domains (File S5). Whitefly Sid1 showed highest similarity with aphids (49–50%) followed by *A. mellifera* (44%). In phylogenetic analysis, whitefly Sid1 was clustered with aphids and result was in agreement with the multiple sequence alignment (Figure 5b). Further, *B. mori* Sid 1, 2 and 3 were clustered together, and closer to the *T. castaneum*.

### Expression analysis of siRNA components

**Figure 6:**
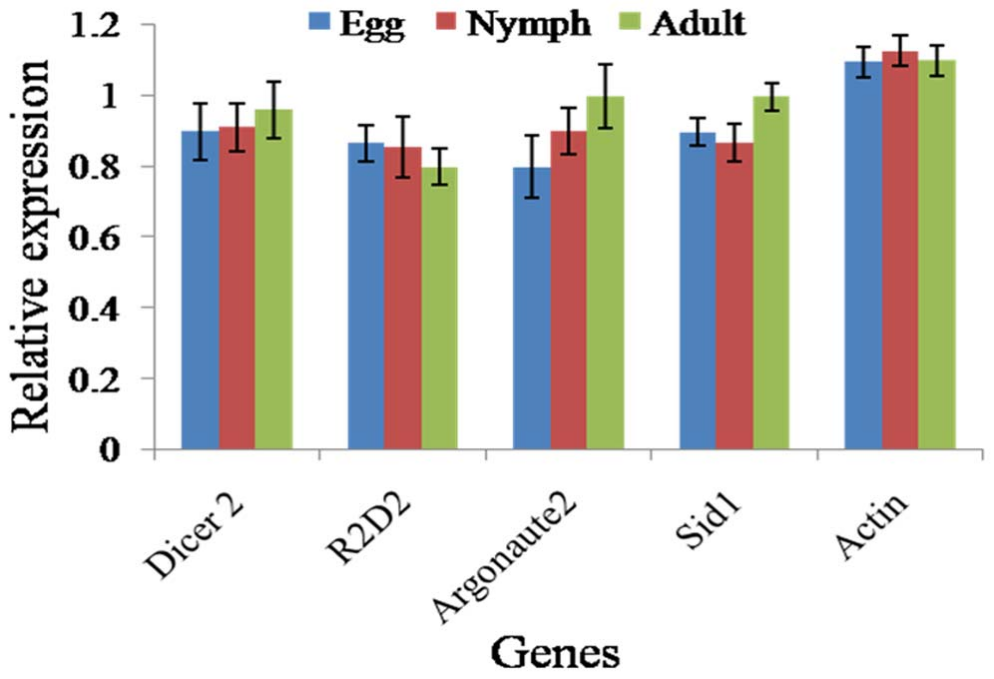
Expression analysis of whitefly *Dicer2, R2D2, Argonaute2* and *Sid1* in egg, nymph and adult insects by real time PCR. Actin is taken an internal control. Figure shows the expression of all four genes in each stage. Further the expression of each gene is comparable and nearby to the expression of *actin*, indicating availability of RNAi components in good quantity in whitefly.

Expression analysis of *dicer2, r2d2, argonaute2* and *sid1* genes of whitefly was performed in egg, nymph and adult insects by real time PCR. Expression level was compared with the *actin* gene. We found that all the genes were expressed at each developmental stage (Figure 6). Significant transcript abundance was observed for each gene which was almost equal to the expression level of *actin*. All the genes expressed at nearly similar level in all developmental stages. Similar result has been reported in case of *A. glycines* and *T. castaneum* [18], [28]. Significant expression of siRNA components in whitefly indicated the possibility of massive siRNA response, and creates a hope for the use of this technique in insect control.

## Conclusion

We observed that the siRNA machinery of whitefly showed significant sequence homology with *aphids* and other insects. Further, transcript abundance of each component was also significant. These results indicated the possibility of massive siRNA response in whitefly. However, the previous reports with whitefly and other insects like *A. pisum* with similar domain organization and expression show inconsistent siRNA response with different target genes. In earlier study we observed that feeding of equal quantity siRNA targeting different genes (actin ortholog, ADP/ATP translocase, α-tubulin, ribosomal protein L9 and V-ATPase A subunit) in whitefly showed diverse kind of responses [24]. Ribosomal protein L9 and V-ATPaseA targeting siRNA caused significant mortality of whitefly in comparison to others. In *A. pisum*, only transient reduction in gene expression is reported after dsRNA injection and feeding [36], [49]. However, injection of siRNAs targeting *coo2* gene of aphid salivary protein showed strong systemic response in *A. pisum* [50]. But similar response was not observed in green peach aphid *M. persicae* for the same gene when delivered through transgenic plants [51]. These variations in RNAi responses might be due to the difference in importance of genes, method of delivery, different role of same gene in various insects and others so many unknown regions. Therefore, future studies regarding the insect control can target multiple genes at a time to get significant response. We have observed in our earlier experiment that the feeding of dsRNA through artificial diet offers the best option for the screening of target gene in insects [24]. Moreover, translation of such technology efficiently in the field by using transgenic plants is necessary [7], [8]. In this process we have developed the transgenic plants expressing the most effective dsRNA (V-ATPase A, which was earlier analysed by feeding in artificial diet) [24] and found similar effect [unpublished data]. Further, present study of characterization and gene expression analysis of siRNA machinery supports our earlier results and opens a new way for the presumption of insect responses towards RNAi.

Sid1 has been reported from diverse groups of insects except some dipterans like *Drosophila* and correlated with the systemic RNAi responses [52], [53]. Further it is highly conserved among different taxa even when they are discrete from each other [18]. Lack of Sid1 in dipteran is astonishing and therefore very deep analysis is required regarding the molecular evolution of Sid1 by wide sampling of insect orders including diptera. Moreover, Luo et al [23] reported that Sid1 is not required for systemic RNAi in the migratory locust *Locusta migratoria*. These reports indicated that wide analysis of different insect is required to reach the base of RNAi.

Systemic and vigorous RNAi response is pre-requisite for the RNAi based pest control using transgenic crops. Knowledge of siRNA machinery and their detail characterization not only explains the molecular mechanism of RNAi, but also indicates the probable response of target insects before developing the transgenic plants.

## Supporting Information

File S1 Complete nucleotide and protein sequences of core RNAi components of whitefly (*B. tabaci*). (**A**) Dicer2, (**B**) R2D2, (**C**) Argonaute and (**D**) Sid 1. Important domains are highlighted by different colours.
(DOCX)

File S2 Sequence alignment of RNAseIIIa (**A**) and RNAseIIIb (**B**) of Dicer2.
(DOCX)

File S3 Sequence alignment of PIWI domain of Argonaute2. Triangle indicates the residues interact with oxygen molecules of 5′P of miRNA/siRNA [ref. 14].
(DOCX)

File S4 Multiple sequence alignment of R2D2.
(DOCX)

File S5 Alignment of Sid1 sequences. Black line denotes the conserved region in N-terminus extracellular domains. Blue lines denote the trans-membrane helix.
(DOCX)

Table S1 Sequences used for various analyses during study.
(DOCX)
