# Supplementary material for: siRNA Machinery in Whitefly (Bemisia tabaci)
Source: PLoS One. 2013 Dec 31;8(12):e83692. doi: 10.1371/journal.pone.0083692 (PMC3877088; doi:10.1371/journal.pone.0083692)
Supplement: File S1 — Complete nucleotide and protein sequences of core RNAi components of whitefly (B. tabaci). (A) Dicer2, (B) R2D2, (C) Argonaute and (D) Sid 1. Important domains are highlighted by different colours. (DOCX) [file pone.0083692.s001.docx]

**Supplementary File 1**. Complete nucleotide and protein sequences of core RNAi components of whitefly (*B. tabaci*)*.* **(a)** Dicer2, **(b)** R2D2, **(c)** Argonaute2 and **(d)** Sid 1. Important domains are highlighted by different colours.

1. **Dicer2 (Accession number: KF740508)**

**Nucleotide sequence**

ATGGAAGGAGCAGCCTCAGGATTTACTCCTCGGGAGTACCAAACATTGATCATGGAAAAGGCAATGAAGGAAAACATCATTATTTACTTGCCTACAGGAGCAGGCAAAACTTATATTGCCTCTTTGTTAATAAAAAGAATGTGCGGGGCAATCAAGAAGCCATTTTCTGAAGGAGGCAAAAGATCCTTTTTCCTTGTGAACACCGTCCAACTTGTAGAGCAACAGGCGCAAGCTGTAAGGCAAGCAATTCCTCTGCGAGTGGGAGAGTACAGTGGAGATAAAGGTGTTGATGGCTGGACGGTGGATCAGTGGAATCAGCAGTTTGATCAACATGAGATTTTGGTCATGACAGCTCAAATTTTCCAAGACTTACTCAGCTCCAATACTATGAAATTAGAAAATGTGAACCTTTTAATATTTGACGAGTGTCACCGAGCTGTTCAGAATCACCCGATGCGTCAGATTATGGAGCAGTTTGAAGGGTTGCCGGTAGAGAAGCAGCCTCGAGTGTTGGGTCTCACTGCGACGCTTTTAAATGGCTCCTGTAAGCCAGAGAATGTAGATGAAGAAATTACTCATCTGGAAAGAACCTTGCAGTCAAAAATAGTGTCGTTTATTGATGATGACCAAGTACTTAGGTTTTCCACAAATCCAAAGGAGCATTTACTCTGCTATGATGATATTGAACGCTGTGACCTTAGCAAATATTGTGATGAACGGATAAAAAAGTTATCAGAAAAAATTTCTCACATCCAATTTGAATTATTTATGCAAAACGTGGAAGCCCCTGTTGATTATTCTCTTTTGACCAATAAGGAGGATAAGCCTAAAAAATGTCTCAAAAATATGCTTGCTGATCTATCTCATGTCCTCAATGACATGAATGTTTTCGGATTATATGTTGGAGCACTAGCGCAAATCGTTCGCTTAGAGAGAATGAAACTGTCCACATGTGATGGATTTTTGTATAATATCTTCAATGTTGTGGTCACAGAGCTAATCTTCATAAGGAAAATCATCAGCGATCACATAGATGAGTCCGATTATGCTTGCGTTTTGAAATACTTATCACCCAAAGTTCTTCTAGTCTTAGATCTTGTCAAAGATAGTGACAAAAACTCAACTTTAATTTTTGTGGAAAGAAGGTTTACAGCGAAAGTGCTGTATTCACTTATCAAGGACTTTATCGTTGAATCTAATCCTAACCTCTTCAAGAACGTTAGGATTGATTTTGTGATAGGAAACACAAACCCTAACATTTTAGCAACCAACGATGAGGCTGTTTTTGCCCGTAAAATTAATTCAGATATCATAGATAAATTTTACCAAAAAAAGTTAAATGTCTTAATAGCCACCGACGTTCTAGAGGAAGGAACGGACATCCCACTCTGCAATGCTGTCATGAAATATGACATCCCTGTCACCTTTCGTAGTTATGTACAATCAAAAGGCAGGGCTAGGTCGAAGGAAAGCAATTTTTATATTCTTGTCCCGAAATGGGACCAAGAAAGATTCTGTATCCGCTACCGCGTTTTTCAAAACATAGAGAAAAAATTAAAAGAGATACTCATCAGAGACCGAATAAAGCCCTCTGAAGAAGAAATTGATGAGGACTTATATCAAGACAGAGAATTACCACCATTTTGTCCCCTTGGCCCGGGCGGACCCAGAGTTACCATGGTAGCTGCTCCGTCCCTTGTTAACAGGTATGTTCAAAGTTTACCGCACGACAAGTTTAGCACGATACTTCCTTTGTACTTTGTCAAACATGAAGTTGAAACAGATGAATACACCGTGAAAATTCAACTGCCATCCAACAGTCCAATTAAAACACTCATTCAGGGCCAACCAATGAAATGTTCCAAGTCAGCTAAGCAAGCAGCTGCATTGGAAGCTTGCAAAGTTCTCTATGAAGCAAAAGAATTGAACCAAAATCTTCTGCCAAGAGGCTCTAGCTCAATTTACCTTGAAGATCCAAAATGGTTTCCTCATTGGAAAGATGAAAAATTTGAAAAAGGCGACCCTGAGCCTGGAACAACAAAAAAAATTAGGATGCACGAGAAAAAGTATTCGACAACCCTTGAAGACTGCCAGCCTGACATTGGTTCGTCAGTATTTTTGCACGTGTTCGATATTAAGCCCAAATTTGAGCTGTCCATAGAGGAGAGTAAGCAGCGAAGGATGTATTATTTCTCCCTCCTGAAAAAAAAGAACACATTTGGCATTCTGACCAACAAGCCCATTCCTCAGATCTGTGATTTTCCATTGTTTATGAAATTTGGTGAATTGGAGGTCAAAATGCGAATGAATATTCCCATAGAAGCACTTAACAAATCCCAGTTGAAACGGCTTCATAAATTTCACTGCCTATTGTTTATGCAAGTGTTAGATGTTGTGAAATCTTTCATGGTGTCTAATGCTACTGAAAGGGCCTCCGTTAATAGTTCCTACATGGTTTGTCCTGTCTATCCAACTGATGAAAATGATCCATCGTCGTACATCATTGATTGGGCAGCAGTTGACAGCTACCAGAAAAAATTCTCAGAGGTAAATGAAGTGAGTGACAGGATTCGCAAAGGAATGTCATTCAACTACGAGTCGCTAGAGGGTTCGCTTATTGTTCCATGGTATCGAGGCTACAACATCTCGAAACAGTCTTACATAGTCACAAGAATAGCCAAAGAATTGAATCCGCTCTCACAATTCCCATCGGTTGAGTACCAGACGTACTCGGATTATTTCAAAAACAAATATTCTAAAACAATTTATCACGACGACCAGTATTTAGTAGATGTGTATCCAGTCAACAGCAAACTTAATTGCATTGTCCCAAGATTAAGAACTAGTAAGCGAAAAATTGATACAGATCGTGAGCACATGGATGAGACTCTCGTACCCGAGCTCTGTGTACAGATAAAATTTCCAACTGTGTATTGGATAAAGGCAATATTCCTTCCCAGTGCCCTGCATCGGCTGAACCGTCTTATGATTTCGGAGGAGCTACGTCTGCGAATAGTTTTGGAAGCTCAAATGGGAGCAGAATTTTATCCACTAGACAAAATTGCTCCCTTAACTGTGGATATAATTAGTGTGGAAAGCCTCCCCCATGTAGAAGCTCCTACTTCACCCGTGAGCACAGTTACTAATTCATTTGAGCGGTCAGTCATGGAGTGGAAAACGGCCAAACGATCGAATGAGTTTCCATGGCTCGACCATGAAATCCCAGCTGATATCGACCGCAATTTGAAAAAAATCTCCATAGTCGATCTGAAAAACTATGATAATTTTTGGGCAAACTCCCCTTCAATCGTCAAGTCTAGGCTCCCAGTTGAAAGTTACCAAAACTCCAAGGTGACTTGCGGTTTAAACACTTGGAGACCCTCCTCAGTCACTCCTTTTCGCTTATCGATTCTTGAAACTTCCTCTTGTAAGCTTGGACCCCAACAGTGCGAGATCTTTCAGTGCATTACATCATTGCAAGCTCATGACATTCAAAATTACGAGTACTTGGAAACACTTGGTGATTCTTATTTAAAGTTTGCTGTCTCATTATCCTTGTTTTTATACTGCGGAGGGATTGATGAGGGCAAATTGACTCGTCTGAAAGGAAAGCTTTTAGGAAACCGCAATCTGTTTTATTGTGCCAATGTTTTGGAGCTGGCTTCCTTGTTAGAGGTTTATTCTTTTTCGCCAAATGATGACTGGTTACCTCCAAGATTTGGTGTGGAAAATACTGTCAGAGAAGTCATGCGTGAGGCAGAGCTCCCACCTGAAGGTTTGTTCCAAATCAATTTCACTGAAGAAGAAAAAATAACTGGTGTGATATCCGAGGAAAAAGATGTTGAAGTAGAAGAGAAATTGTTTGAACTTGCCTCTGCAGCTGCAAACGAAGAAGAACCTCGAGGACGCTCTCCTTGTTCAATTTTGTACTTGAATCGCCAAGAAGTGCAGGATAAGAATGTTGCTGATTGTGTCGAAGCCTTGTTGGGAATTTATGTAAAGAAATGCGGTATTCAAGCAGCTTTTCGAATGCTCTTATATTTCGGGATTTTGCCTGACTCAATCATACGCCAAGACCTTGTTTTTGACGATACTTTTGTCCCAGACGTAAGCATGAATCCAGCAGCTGATACCCCACAGCAAGTGGAGTTCCTTTTACAAGAATCCTGTTACGAAGTATTAGAAAGGCAAATTGGATACACGTTTAAGGACAAGAGATATATTCTACAAGCTTTGTCTCACACTTCCTTTACTCAAAATGTGATCACTGAATGTTATCAACGCCTGGAATTTCTAGGGGATGCAATTCTTGACTTTCTAATCACGTGCTATATCTTCGAAAAAGGTCAACATCTAACTCCAGGGCAAGTGACTGATGTCCGATCAGCCTGTGTGAATAATGTCACTTTTGCATGTTTAGCTGTGCGGTATAGCATGCACAAATCTCTCCTATGCAGATCTGTGACTCTGATCAATGAAATTGACAAGTTTGCAAAATATCAGGAAGGTAAAAACCACAGAATTGGAGAAGAGGTTTTAATTTTGTTAGCTGAGGATGATCTAAAAATCGCTGAGGCCATTGATGTCCCAAAGGCTCTCGGAGATATTTTCGAATCTATTGCTGGAGCAATTTACCTTGATAGTGGCAAAGATTTGAAAACTGTCTGGAGGGTGTATTACAACTTGATGCATAAAGAATTAGAGGAATTTTGTAATAAGCCACCAAAGAACGTAGTGCGCAAGCTGCATGAATTGAACATAGATGCAAAATTCATGCCTTCAGAACCAGTTCCTGATGTTGCAGACATTGTTATGGTGCCTTTGGAGGCGCGTATAAACAATGAAATGAAGAAAGTGTTTGGTTTTGGGCGCAACAAATCTGCTGCAAAGAAGGCTGCTGCCAAAATGATCACCAACATAGTGATTGCCAATTCCAATGCTTAG

**Protein sequence** (Highlighted domains are-Helicase: yellow, PAZ: green, RNAseIII: blue)

MEGAASGFTPREYQTLIMEKAMKENIIIYLPTGAGKTYIASLLIKRMCGAIKKPFSEGGKRSFFLVNTVQLVEQQAQAVRQAIPLRVGEYSGDKGVDGWTVDQWNQQFDQHEILVMTAQIFQDLLSSNTMKLENVNLLIFDECHRAVQNHPMRQIMEQFEGLPVEKQPRVLGLTATLLNGSCKPENVDEEITHLERTLQSKIVSFIDDDQVLRFSTNPKEHLLCYDDIERCDLSKYCDERIKKLSEKISHIQFELFMQNVEAPVDYSLLTNKEDKPKKCLKNMLADLSHVLNDMNVFGLYVGALAQIVRLERMKLSTCDGFLYNIFNVVVTELIFIRKIISDHIDESDYACVLKYLSPKVLLVLDLVKDSDKNSTLIFVERRFTAKVLYSLIKDFIVESNPNLFKNVRIDFVIGNTNPNILATNDEAVFARKINSDIIDKFYQKKLNVLIATDVLEEGTDIPLCNAVMKYDIPVTFRSYVQSKGRARSKESNFYILVPKWDQERFCIRYRVFQNIEKKLKEILIRDRIKPSEEEIDEDLYQDRELPPFCPLGPGGPRVTMVAAPSLVNRYVQSLPHDKFSTILPLYFVKHEVETDEYTVKIQLPSNSPIKTLIQGQPMKCSKSAKQAAALEACKVLYEAKELNQNLLPRGSSSIYLEDPKWFPHWKDEKFEKGDPEPGTTKKIRMHEKKYSTTLEDCQPDIGSSVFLHVFDIKPKFELSIEESKQRRMYYFSLLKKKNTFGILTNKPIPQICDFPLFMKFGELEVKMRMNIPIEALNKSQLKRLHKFHCLLFMQVLDVVKSFMVSNATERASVNSSYMVCPVYPTDENDPSSYIIDWAAVDSYQKKFSEVNEVSDRIRKGMSFNYESLEGSLIVPWYRGYNISKQSYIVTRIAKELNPLSQFPSVEYQTYSDYFKNKYSKTIYHDDQYLVDVYPVNSKLNCIVPRLRTSKRKIDTDREHMDETLVPELCVQIKFPTVYWIKAIFLPSALHRLNRLMISEELRLRIVLEAQMGAEFYPLDKIAPLTVDIISVESLPHVEAPTSPVSTVTNSFERSVMEWKTAKRSNEFPWLDHEIPADIDRNLKKISIVDLKNYDNFWANSPSIVKSRLPVESYQNSKVTCGLNTWRPSSVTPFRLSILETSSCKLGPQQCEIFQCITSLQAHDIQNYEYLETLGDSYLKFAVSLSLFLYCGGIDEGKLTRLKGKLLGNRNLFYCANVLELASLLEVYSFSPNDDWLPPRFGVENTVREVMREAELPPEGLFQINFTEEEKITGVISEEKDVEVEEKLFELASAAANEEEPRGRSPCSILYLNRQEVQDKNVADCVEALLGIYVKKCGIQAAFRMLLYFGILPDSIIRQDLVFDDTFVPDVSMNPAADTPQQVEFLLQESCYEVLERQIGYTFKDKRYILQALSHTSFTQNVITECYQRLEFLGDAILDFLITCYIFEKGQHLTPGQVTDVRSACVNNVTFACLAVRYSMHKSLLCRSVTLINEIDKFAKYQEGKNHRIGEEVLILLAEDDLKIAEAIDVPKALGDIFESIAGAIYLDSGKDLKTVWRVYYNLMHKELEEFCNKPPKNVVRKLHELNIDAKFMPSEPVPDVADIVMVPLEARINNEMKKVFGFGRNKSAAKKAAAKMITNIVIANSNA

1. **R2D2 (Accession number: KF740509)**

**Nucleotide sequence**

ATGAGTCTCAGCAATATTGGACAAAAAACGGCAGCCTCCATTTTACAGGAGCACTTGATGAAGTTGGGGCATGTTCCACAATACGATTTAGTCCGTGATGATACTGGTACGCATATTCCCACTTTTACTTACCGACTAGAGTTTAATAAGGTTGTTGTTGAGGGAACTGGTTCGTCCAAGAAAGAAGCAAAACAGACAACAGCTCGTTTGATGTTAGAAAAACTAGGAATAACCAGTGCGGAAGAAATGCCAAAAATTGAGCCTACTGAGGCCTTACCCATGAAGAAAGTGTGCAAGTATAATTCTGTTGGTGCATTAACTGAGTTCTGCGTCCAAATGAAAATGGCTCAAGGTCCACAGTACTTCGATGTGAGAGAGGAGGGCCCGCCCCATGCTAAATTGTTTACTGTCAGATGCAGTGTATCCCTCCTGTCAGAAACAGCAGTTGCTGGGACGAAAAAGCAAGCAAAGCAACAAGCAGCTCAGCAAATGTTGACAAGGTTGAAGAACCTTCAAGTTATTGATGACGAGAACCAAATTCTTTCGCCAGGCTCTTTTGAAGTAGTGACACCTGAAGACGAACAGCCTTTGCTCAGTGACGATTTAAAGCAGAGAGTACTGGATCACTTTAAAGCCCTCCACTCATTTACATCACAGCAAGTTTGTGGCAGCAGTGTATCAAAATACCATACCGTATTTAAATCAGAGAACACCACTGCATCAGAAGTTATTAGTAATCTAGTATCTCGGGTCAGAAATCCTGACTCTCCTCTCAATTACAACTTACAGGCACTTCAAGAGGGTAGAGAGGAACTTTTCAAAGAAGTTATTTCGGAGTTAAATGTTGAGGAGACATTCATTGAGATTCCTGATGAAAAAAACCCTCAAATTCTTCTTAATGTTAGCCATGCACCTAATGTTTCCTTTCTAGCCTGTGCATCTTCCAAAGAAATAGCCAGGAAAATTGCTATTACTCAGTATTTACAATTTATCTCTACTTTAGTTTCATAA

**Protein sequence**(Highlighted domains are-DSRBD: yellow)

MSLSNIGQKTAASILQEHLMKLGHVPQYDLVRDDTGTHIPTFTYRLEFNKVVVEGTGSSKKEAKQTTARLMLEKLGITSAEEMPKIEPTEALPMKKVCKYNSVGALTEFCVQMKMAQGPQYFDVREEGPPHAKLFTVRCSVSLLSETAVAGTKKQAKQQAAQQMLTRLKNLQVIDDENQILSPGSFEVVTPEDEQPLLSDDLKQRVLDHFKALHSFTSQQVCGSSVSKYHTVFKSENTTASEVISNLVSRVRNPDSPLNYNLQALQEGREELFKEVISELNVEETFIEIPDEKNPQILLNVSHAPNVSFLACASSKEIARKIAITQYLQFISTLVS

1. **Argonaute2 (Accession number: KF192313)**

**Nucleotide sequence**

ATGTCCAAAAAATCTCCCTCTCACCAACCATTGTTTCCAATTCCTCATCGCAAAGGAGTAGGTACCTTAGGGAGGCGAACACAGGTAGAAGTAAATCAATTACCATTAAATCTAAGCAAGTTATGCCCTGAGATCTATCACTATGATGTGGCTGTAGACCCAGACAAACCAAAAAGATTTCTTAGACCTATTGTTGCCGAATTCATTAAAAGACATTGTAATGGCTTTAATCCTGCGTTTGATGGGAAGAAAAATCTGTACGCTGCTAGACAGCTGCCTTTTGGCACTTCAAAAAGTGATCAAATTACCATGACAAACGAAGAGAGAGTATCGAATAATGAGCTTGAATTTCAAATCACAGTGAAGTTAGCTCAAAAAATCAACACAAGCACCATAACAGCGTTCTTGAACTCAAGAACGACTGGGCAGCGGTTGGATGTGCCGCAAACAGCAATTCAAGCAATTGATGTAGCTCTGAGAGCTGCGCCGTTAGTTTCTGGTCCTGTCCCTGTTGGCAGAAGCTACTTTATCCCTCCAGATGGAAGAGAACGAATTATTGAGCTAGGAGGTGGACTTGAACTCTGGTATGGCTTTTATCAGTCCGCCATTCTAAACTGGAAACCCTTCTTGAATGTCGACGTTGCTCACAAAGGCTTCCCTATTCCTACCAATTTAGTCGAGATTTTCTGTCAAGTTTGCAGATTGAACAATCCAAGGGACAATTTCCGACCTGACGATGTATTAAAGTTCAGAGGATTTGTCACAACTCTTAAGTGTGATTATGAAATACCTAACACTCCAAATTCAAGAAGAACGTATCGAATCAACAATGTCGGAAAGAGTCCTGCAAACCTCAGATTCCGTCCGGACAATGGACCAGAAATATCAGTTGTTGATTATTTTAAAAGAGTGAAGAATTATCAAATCAAGCACCCAGACTTGCCAACTGTGCAAGTAGATCCACCTGCAAAAAATATTTTCCTACCAATAGAGTTGTGTTACCTGAAACCTGGTCAGGCTTTAAATCGAAAATTAGATGAGGAGCAAACTGCAGCCATGGTCAAAGCTGCTGCAAAACCACCAGAAGACCGAAAAAGAAGGATTCTCAATGCAATCCGCACTGCTCAATACAATAGATCACCAGTTGTTAAAGAATTTGGTATTGAGGTTAACGAAAATCTAGATAAAGTTGACGCACGAATTCTGGACCCTCCAGGTGTTCAGTACAGAAATCAATCCAATCCGAGGCCACAGCCTGGACAATGGCGTGCAGGAGAATTCATGTTAAGCCATGAGCTTTCAAAATGGTTCATTATGTGTCTAGACTTCCGCACCAGGCGAGAAAAGCTCAATGATTTTGCTAGAATGCTTGCGTCACAGGGCAGGAATTTTGGCATGACCATCCAAAATGTCACTGAAATAAAAGAAATGGATACCCGCAGCAGGCAAATTAAAGCTGATGTTACTAATGTCCTTCACAAATGGAAAGCCCAAGGTGCAGAATTGGTAGTTGTTGTCATACCTGGTCACGGCGATTTTTACAGTATGGTAAAACGATGTGCTGAGCTTGAAGTAGGTGTTTTAACTCAATGTATCAAGGCCAATACAATGTTCAAAATGAATCCTGCTACCTGTGGGAATATCCTTTTGAAAGTAAATTCTAAAACTAATGGTAAGAATCATCAGCTAGGAGATCGATACAAACCTGGCGTTCTGAGTCGTCCTGTAATGTTGATTGGAATCGACGTTACTCATCCATCTCCAGACCAAACTTCAATACCGTCCGTTGCTGCTGTTGCTGCCTCTCATGATGCTACAGCATTCCAGTACAACATGATATGGCGACTGCAAAATCCAAGAGAAGAAATCGTTGTTGATCTGAAAAACATCATCATTGAGCAATTGAAGTTCTTCTTCACAAAGACCCGTTATAAACCTGAAAAAATTATTGTCTACCGAGATGGAGTCAGTGAAGGCCAATTTCAACAGGTTCTCGCTGCGGAATTGAATGCTATCAGACAAGCATGTACTACTCTGGAGAAGGACTACAAGCCAGGAATAACATTTTTGGTCGTACAAAAGCGTCATCATGTTCGATTTTTCCCAATGAAAAGCCAAGATGAAGATGGGAAAAATAGGAATGTTCCTCCTGGCACTATTGTGGATACCACTATCACACACCCAAGGGAATTAGATTTTTATCTTGTATCCCATTCCAGTTTACAGGGTACATCACGGCCAACCAAATATCATAGACTCTGGGACGACAATAACATCTCTGAGGATGAATTGGAAGTCTTGACCTACTATCTGTGTTACCTCTTTTCACGTTGTACTCGATCTGTGTCGTATCCTGCGCCAACTTACTATGCTCACTTGGCAGCTTTTAGAGCTCGAACTTATCTAGAGAACAACCCGGCCCCCTTGAATAACTTGGAAGGATTTGCATCCAAAAACAAGATCGAGCCAGTTTTCATGAAAAATACCCCAATGTTCTTTGTTTAG

**Protein sequence**(Highlighted domains are-PAZ: yellow, PIWI: green)

MSKKSPSHQPLFPIPHRKGVGTLGRRTQVEVNQLPLNLSKLCPEIYHYDVAVDPDKPKRFLRPIVAEFIKRHCNGFNPAFDGKKNLYAARQLPFGTSKSDQITMTNEERVSNNELEFQITVKLAQKINTSTITAFLNSRTTGQRLDVPQTAIQAIDVALRAAPLVSGPVPVGRSYFIPPDGRERIIELGGGLELWYGFYQSAILNWKPFLNVDVAHKGFPIPTNLVEIFCQVCRLNNPRDNFRPDDVLKFRGFVTTLKCDYEIPNTPNSRRTYRINNVGKSPANLRFRPDNGPEISVVDYFKRVKNYQIKHPDLPTVQVDPPAKNIFLPIELCYLKPGQALNRKLDEEQTAAMVKAAAKPPEDRKRRILNAIRTAQYNRSPVVKEFGIEVNENLDKVDARILDPPGVQYRNQSNPRPQPGQWRAGEFMLSHELSKWFIMCLDFRTRREKLNDFARMLASQGRNFGMTIQNVTEIKEMDTRSRQIKADVTNVLHKWKAQGAELVVVVIPGHGDFYSMVKRCAELEVGVLTQCIKANTMFKMNPATCGNILLKVNSKTNGKNHQLGDRYKPGVLSRPVMLIGIDVTHPSPDQTSIPSVAAVAASHDATAFQYNMIWRLQNPREEIVVDLKNIIIEQLKFFFTKTRYKPEKIIVYRDGVSEGQFQQVLAAELNAIRQACTTLEKDYKPGITFLVVQKRHHVRFFPMKSQDEDGKNRNVPPGTIVDTTITHPRELDFYLVSHSSLQGTSRPTKYHRLWDDNNISEDELEVLTYYLCYLFSRCTRSVSYPAPTYYAHLAAFRARTYLENNPAPLNNLEGFASKNKIEPVFMKNTPMFFV

1. **SID1 (Accession number: KF192314)**

**Nucleotide sequence**

ATGGAAATCAACAAGACTGTCTCATATCTCTTTGAATATCCAAATTACAAGGATGTAAGTGACTTGGAGCCTCCTCGGGTGACCATCAGTAGTACTAGTGCCGACGAATCATACCCAATTATAATCGTAGTCCAGCAGAGAAGAAGTGTTTTATCTTGGAAGCTTCCTCTCACTATTCAGTCGAAACAAAATAGTTATGCATATCATCGTACATCTAGGACACTATGCACTGATTTAGAGGATGTACAAAACCCAGAAGGCGAGCATGAAATGGTCATTAGTGTCTCCACATCCAGTCCGTCCAATGTTAATTTCTCTCTGGAAGTTGTAAAGCAACAAAATTTCAGCATCAAATTGGAGGATGAATATGAAACAGTAATCACACCTTCAGAGCCAGCATTCTTCCTTTACAACTTTAGTGAAAACGTATCATCTGTTCTTCTTGAAATCGACTCTCCAGATAAAACGTGCATGACCCTATCCATCCAAAACATATCATGTCCCGTTTTTGATCTGGAACACACAATTCAGTACAGAGGAGATTGGGAAACGGTCAGTTCCAAAGGAGGAATTATGCTGACGCGGCAGGATTTTCCTGAGGGTCTGTACATTGTATTTGATCTCCACAGTGATGATTCAGACTGCATATCAAGCACTGTTGATGGTCCAGAGGTAGATGTATCAGAGGCAGATAGGAAGAAAGTGATCCGTTTTAAATTGAACAAAAATCTAAACTACGAAGATTACCTATTTGCATCTCTGGCGGTTTTACTCTCATTTTCCTCCGTCTTTATACTTTCAGGCCTTTTACTGTGTTGTACCAGGAAGAGCCCGGAAACTGTAACCGTCTCAGAAGAGTCTTGCACAGTCATTTCAGCCGGTGAAATCATTAATCCAAATGATTCTGTGAGTGCAGATTCCTCTCTAGATGAAGAAGAAACTGATAAGAGCAGTAAAGAAAGCAAAGAATTAGTTGGAACAAAAACATTTTTATTCGTGAGCGATCTGTCGAAAAAAAATCCTCAAGCTGTGCAGGCTAAAGCTCGGTTGTACTTTTGGAACCTCCTTACTGTTGCTGTATTTTACTCACTTCCTGTTGTGCAACTAGTCTTCACCTATCAAAAGGTGTTGAACATGTCCGGGAATCAGGACATGTGCTACTACAATTTTCTTTGCTCACATCGGCTTCTGCAAATAAGCGACTTTAATCATGTCTTCACCAACATTGGGTACATATTGCTTGGTCTACTTTTTATACTACTTGTATATCGCAAGGATGCAGCCTGCCAGCTCCACTCTACAAAAGGTATTCCTCATCATTTTGGACTGTACTATTCCATGGGAACTGCTCTGATGATGGAAGGGATTCTAAGTGCTTGCTATCACATTTGTCCAAATCATTCAAATATTCAGTTCGATACAAGTTTCATGTATATTATTGCCATGTTATCCATGCTAAAAATCTACCAAAATCGACATTCTGATATCAATGCGAGTGCATACACCACTTATCTAGTCCTTGCTTGTGTGATTTTCATCGGAATGTGCGGTATTCTCAATGGCTCCTTCATATTCTACGTTGTTTTCACTGCACTCCACATCATTACATGTTTTTTTCTATCACTTCAAATCTACTACATGGGTCGATGGAAACTAGGATTTCATTCTTTCAAGAGGACTATTATCGAATTCTTCACGAATCTCCGAGCTGGACTGAGGCACTGTAAACCCATGTATCCCAATCGTATGGTTCTTCTAATTTTAGGAAACGCCTGTAATTGGGGGCTAGCTGTGCATCTCTGGATGTCCAACCGAAGCAATTTTGCAACGTATTTGTTGATCATATTCATGGCAAATTTAATTTTATATTTAACTTTTTACATCACCATGAAACTACTATCTGGTGAAAGGATTCTCCTACAACCCTTCCTGTACATAATTTTTGCCGTCATTTTTTGGGGTGCCTCTGGATACTTTTTTATGAGCAGATCAACTAACTGGCAGTTAACACCTGCTGAATCTAGAACCTACAATAAATCTTGTATGTTGCTGAAATTTTACGACAATCATGACATCTGGCACCTGATATCTGCTGGTAGCATGTTTTTCTCATTCATGGTTCTTCTCACTTTAGATGACGATCTGAAAGATAAAGAGCGGAAATTGATTCCTGTGTTTTAA

**Protein sequence** (Domain organization in given in **Supplementary File 5**)

MEINKTVSYLFEYPNYKDVSDLEPPRVTISSTSADESYPIIIVVQQRRSVLSWKLPLTIQSKQNSYAYHRTSRTLCTDLEDVQNPEGEHEMVISVSTSSPSNVNFSLEVVKQQNFSIKLEDEYETVITPSEPAFFLYNFSENVSSVLLEIDSPDKTCMTLSIQNISCPVFDLEHTIQYRGDWETVSSKGGIMLTRQDFPEGLYIVFDLHSDDSDCISSTVDGPEVDVSEADRKKVIRFKLNKNLNYEDYLFASLAVLLSFSSVFILSGLLLCCTRKSPETVTVSEESCTVISAGEIINPNDSVSADSSLDEEETDKSSKESKELVGTKTFLFVSDLSKKNPQAVQAKARLYFWNLLTVAVFYSLPVVQLVFTYQKVLNMSGNQDMCYYNFLCSHRLLQISDFNHVFTNIGYILLGLLFILLVYRKDAACQLHSTKGIPHHFGLYYSMGTALMMEGILSACYHICPNHSNIQFDTSFMYIIAMLSMLKIYQNRHSDINASAYTTYLVLACVIFIGMCGILNGSFIFYVVFTALHIITCFFLSLQIYYMGRWKLGFHSFKRTIIEFFTNLRAGLRHCKPMYPNRMVLLILGNACNWGLAVHLWMSNRSNFATYLLIIFMANLILYLTFYITMKLLSGERILLQPFLYIIFAVIFWGASGYFFMSRSTNWQLTPAESRTYNKSCMLLKFYDNHDIWHLISAGSMFFSFMVLLTLDDDLKDKERKLIPVF
