# Supplementary material for: siRNA Machinery in Whitefly (Bemisia tabaci)
Source: PLoS One. 2013 Dec 31;8(12):e83692. doi: 10.1371/journal.pone.0083692 (PMC3877088; doi:10.1371/journal.pone.0083692)
Supplement: File S2 — Sequence alignment of RNAseIIIa (A) and RNAseIIIb (B) of Dicer2. (DOCX) [file pone.0083692.s002.docx]

**Supplementary File 2.** Sequence alignment of RNAseIIIa (a) and RNAseIIIb (b) of Dicer2.

**(a)**

**
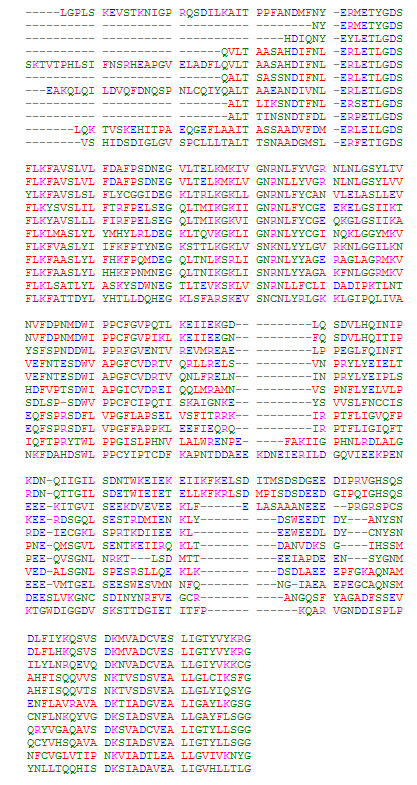
**

A. glycines (45%)

A. pisum (44%)

B. tabaci

L. striatella (42%)

N. lugens (40%)

B. germanica (38%)

T. castaneum (36%)

D. plexippus (40%)

B. mori (39%)

D. melanogaster (30%)

C. elegans (25%)

**(b)**

**
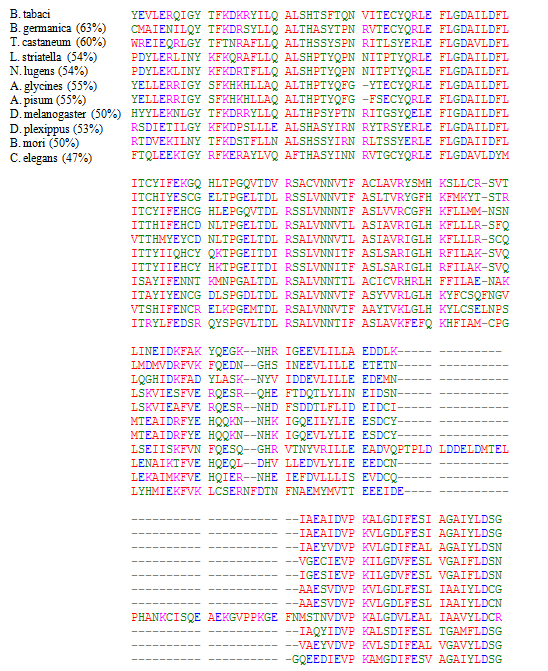
**
