# Supplementary material for: siRNA Machinery in Whitefly (Bemisia tabaci)
Source: PLoS One. 2013 Dec 31;8(12):e83692. doi: 10.1371/journal.pone.0083692 (PMC3877088; doi:10.1371/journal.pone.0083692)
Supplement: File S3 — Sequence alignment of PIWI domain of Argonaute2. Triangle indicates the residues interact with oxygen molecules of 5′P of miRNA/siRNA [ref. 14]. (DOCX) [file pone.0083692.s003.docx]

**Supplementary File 3**. Sequence alignment of PIWI domain of Argonaute2. Triangle indicates the residues interact with oxygen molecules of 5’P of miRNA/siRNA [ref. 41].

B.tabaci -LVVVVIPGH-GDFYSMVKRCAELEV--GVLTQCIKANTMFK-MNPATCG

N.Lugens(71) -IVIVVIPEQ-AEIYALVKQTAELSV--GILTQCIKSKTMYK-MNPATIG

T. castaneum (61) -LIIVVVPNS-GPQYSLVKQAAELNV--GCLTQCIKERTIAK-LNPQIIA

T. castaneum (63) -LIIVVVPNS-GPQYSFVKQAAELNV--GCLTQCIKERTIGR-LNPQTVG

A.mellifera (59) -LVVVILPNL-DNAYSIVKQISELQIHEGIVTQCIKNQTLKK-LNDSTIG

L.migratia (65) ELAVVVIDDK-RADYAQVKQFAELQV--GVLTQCLKARTLSR-YNYSTVQ

A.glycines(65) -LIVVIIPDAPAGVYGMVKQTSELEI--GVLTQCIKSRTMFK-MNPSTSS

A.pisum(64) -LIVVVIPDYPAGIYASVKQKSELEV--GILTQCIKSKTMFR-MNTSTSS

B.mori(49) -FLVVVVSGRGRDYYHKLKQIAELKV--GILTHVFKEDTATRRMNPQTAR

D.melanogaster(48) -LAIVIIPQF-RISYDTIKQKAELQH--GILTQCIKQFTVERKCNNQTIG

C.elegans(50) -LIVVVLPGK-TPIYAEVKRVGDTVL--GIATQCVQAKNAIR-TTPQTLS

NILLKVNSKTNGKNHQLGDRYKPGVLSRP-VMLIGIDVTHPSPDQTSIPS

NILLKVNSKLNGLNHKLGGR--PKLLARP-AMIMGADVTHPSPDQVNIPS

NILLKINSKLNGTNHILS--SRLPIMSRP-CIIMGADVTHPGPDAKDVPS

NILLKINSKMNGTNHRLSPNSRPLIMKRP-CMIMGADVTHPSPDARDIPS

NILLKINSKLNGINHIITPTNRPNCLYQP-CMIIGADVTHPSPDATNIPS

NILLKVNSKLNGVNHNLAPPTRPPCLHKP-IMIIGADVTHPSPDQRNIPS

NILLKINSKLNGVNHTLAIKSSPPSMDG--AIIFGADVTHPSPDQTAIPS

NILLKINSKLNGINHTLAIRSSPPSMEG--AIIFGADVTHPSPEQTTIPS

NILLKVNSKLMGINQALENRSIPQCLKGGAVMIVGADVTHPSPDQSNIPS

NILLKINSKLNGINHKIKDDPRLPMMKN--TMYIGADVTHPSPDQREIPS

NLCLKMNVKLGGVNSILLPNVRPRIFNEP-VIFLGCDITHPAAGDTRKPS

VAAVAASHDATA-FQYNMIWRLQNPREEIVVDLKNIIIEQLKFFFTKTRY

VAAVSASHDANG-FMYNMMWRLQPAKTEIIEDLQAIVVAQLKYFFQKTRC

VAAVTASHDPNA-FQYNICWRLQPPKVEIIEDLCAITVEQLMFFYRKTRH

VAAVTASHDPNA-FQYNICWRLQPPKVEIIEDLCNITVEQLKFFYQKTGF

IAAVAASHDPNA-FKYNVEIRLQSPREEIIQDLEEIMIIQLKYFYVTTGQ

VAAVCASHDPDA-FQYNIQYRLQPPRMEIIQDLAEIMRNHLIFFYKATGH

VAAVAASHDIFG-SQYNMEWRLQSPKVEIIQDLEDIVHIQLLKFKEKTKA

VAAVAASHDTYG-SQYNMEWRLQSPKVEIIQDLEDIVHIQLLKYKERTKT

IAAVTASMDTKC-YIYNIELSIQTPKKEMIVQFEDIMVDHFHAFKKSQGI

VVGVAASHDPYG-ASYNMQYRLQRGALEEIEDMFSITLEHLRVYKEYRNA

IAAVVGSMDAHP-SRYAATVRVQQHRQEIITDLTYMVRELLVQFYRNTRF

KPEKIIVYRDGVSEGQFQQVLAAELNAIRQACTTLEKD-YKPG--ITFLV

KPETIYFFRDGVSEGQFNQVLSAELTAIRKACRTLQED-YKPG--ITFLV

KPETIVFFRDGVSEGQFAEVRRAEISAIHQACKKLQREGYEPR--ITFLV

KPESIVFFRDGVSEGQFKQVQRAEIAAIQKACKMLQKDDYEPK--ITFLV

KPQKLIFYRDGVSEGELVKIMHKELSAIKRAIARLEKS-NELRIPITFLV

KPHRLIFYRDGVSEGQFAMVLSEELSQIRNACRGLEEL-YEP--PITFLV

IPKKIFYFRDGVSEGQFLQLLEYELIAIRRACLRLNIN-YTPS--VTFLV

VPKKIFYFRDGVSEGQFLQLLEYELIAIRRACLRLNIA-YKPS--VTFLV

LPKKVFVFRDGVSEGQFAEVMKSELTGLHRAYQRVAGLNAKPE--VLFIL

YPDHIIYYRDGVSDGQFPKIKNEELRCIKQACDKVG-CKPK----ICCVI

KPARIVVYRDGVSEGQLFNVLQYELRAIREACVMLESG-YQPG--ITFIA

VQKRHHVRFFPMKSQDEDGKNRNVPPGTIVDTTITHPRELDFYLVSHSSL

VQKRHHTRFFPKHDRDKEGKFGNVPAGTIVDTQICHKSETDFYLVSHASI

VQKRHHTRLFPTNPRDSEDRNNNVPAGTCVDTHITNPMMQDFYLVSHASI

VQKRHHTRLFPTNPRDSEDKNNNVPAGTCVDTHITNPRMQDFYLVSHASI

VQKRHHVRFFPTDAKNSDDKNFNVQAGTIVDTEITHPTHIDFYLVSHASI

VQKRHHTRFFPKRKEDEDGKNKNVPAGTIVDTDITHPRHIDFYLVSHASI

VQKRHHTRMFPKCSIDMDGKFGNVPSGTIIDTQITHPTELDFYLCSHASI

VQKRHHTRMFPKFSYDMDGKFSNVPSGTIIDTQITHPTELDFYLCSHASI

VQKRHHTRFFLPGNN---ARFN-VDPGTVVDRDIVHPRELDFYLVSHQAI

VVKRHHTRFFPSGDVTTSNKFNNVDPGTVVDRTIVHPNEMQFFMVSHQAI

VQKRHHTRLFAADKADQVGKAFNIPPGTTVDVGITHPTEFDFFLCSHAGI

QGTSRPTKYHRLWDDNNISEDELEVLTYYLCYLFSRCTRSVSYPAPTYYA

QGTARPTKYHLLWDDNDIDEDDLEELTYSLCHLFTRCTRSVSYPAPTYYA

QGVAKPTKYCTLWDDNNMSNDDIEELTYYLCHMFTRCNRSVSYPAPTYYA

QGVAKPTKYCTLWDDNNMNNDDIEELTYHLCHMFTRCNRSVSYPAPTYYA

QGTARPTKYRCICNENQMPENEIEELTYYLCHMFARCTRSVSYPAPTYYA

QGVSRPTKYRTLWDDSHMSMDELEELTYYLCHLFSRCTRSVSYPAPTYYA

QGTSRPTKYHLIWDDNNFTEDQLEQLTFYLCFMFARCTRSVSYPAPTYYA

QGTSRPTKYHLIWDDNNFTEDQLEQLTFYLCFMFVRCTRSVSYPAPTYYA

KGTARPTRYHAVCNDGRIPENEVEHLAYYLCHLYARCMRAVSYPAPTYYA

QGTAKPTRYNVIENTGNLDIDLLQQLTYNLCHMFPRCNRSVSYPAPAYLA

QGTSRPSHYHVLWDDNDLTADELQQLTYQMCHTYVRCTRSVSIPAPAYYA

HLAAFRARTYLE

HLAAFRARVYLE

HLAAARAKVYVE

HLAAARAKVYIE

HLAAFRARALIH

HLAAFRARMYYE

HLAAFRARAYIE

HLAAFRARAYIE

HLACLRARSLTY

HLVA------ --

HLVAFRARYHLV
