# Supplementary material for: siRNA Machinery in Whitefly (Bemisia tabaci)
Source: PLoS One. 2013 Dec 31;8(12):e83692. doi: 10.1371/journal.pone.0083692 (PMC3877088; doi:10.1371/journal.pone.0083692)

**Supplementary File 4**. Multiple sequence alignment of of R2D2.

A. glycines (28%)

A. pisum (22%)

B. tabaci

A. mellifera (26%)

P. humanus (27%)

T. castaneum (19%)

D. melanogaster (15%)

B. mori (22%)

C. elegans (20%)


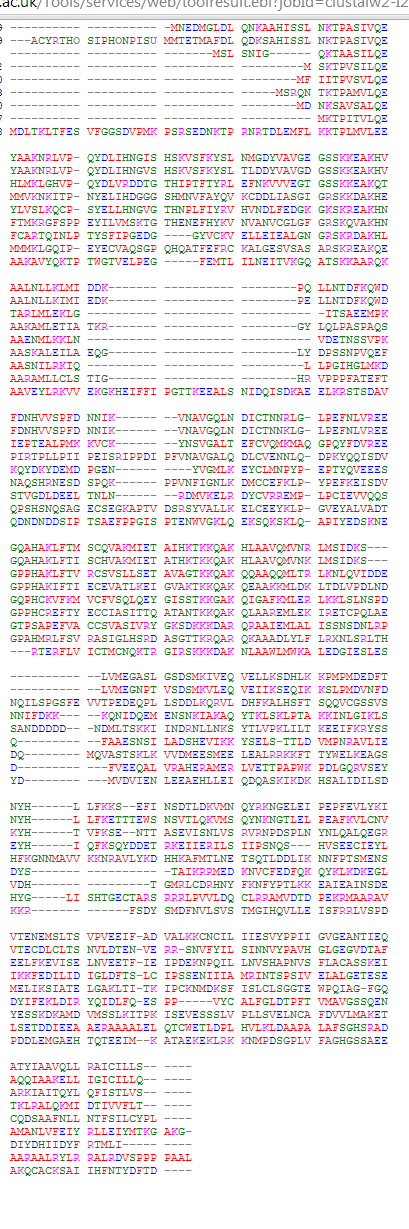

Supplement: File S4 — Multiple sequence alignment of R2D2. (DOCX) [file pone.0083692.s004.docx]
