# Supplementary material for: siRNA Machinery in Whitefly (Bemisia tabaci)
Source: PLoS One. 2013 Dec 31;8(12):e83692. doi: 10.1371/journal.pone.0083692 (PMC3877088; doi:10.1371/journal.pone.0083692)
Supplement: File S5 — Alignment of Sid1 sequences. Black line denotes the conserved region in N-terminus extracellular domains. Blue lines denote the trans-membrane helix. (DOCX) [file pone.0083692.s005.docx]

**Supplementary File 5**. Alignment of Sid1 sequences. Black line denotes the conserved region in N-terminus extracellular domains. Blue lines denote the trans-membrane helix.

B. mori_1(39) ----------MMGYRKILLLMLIKISYCFKNSVNLAVNRTFQYNIYNYDT

B. mori_2 (37) --------------------------------------------------

A.glycines(49) -------MWKPIFVIIFGLSSVWSELFSENVERYGSNDLIPIVLKGNYSQ

A.gossypii(49) -------MWKPIFVVIFGLSSVWSELFSENVERYGSNDPIPIVLKGNYSQ

A.pisum(50) -------MWKPILVVIFGLSSVWSELISGQYASYKSNDLIPIVLKGNYSQ

B.tabaci --------------------------------------------------

N. lugens(43) MIFSILFKMRNVLVYYVFLSFISTAVLSLNFESILKNSENATVFHYCEFN

A.mellifera(44) ---------------------------------------------MANYT

T.castaneum_3 (43) -----------------MTPKMLHLFLIMSAVTVICDSFNPIYLNLSYSN

T. castaneum_1(36) -----------------MIAAAGLLLLVPLADCAHIASLNIEQHQGNYSQ

T.castaneum_2 (30) -----------------MATSWFFVAIVPLVLCLQPKIVMVPQFG-RVSQ

B. mori_3 (32) --------------------MISWCALALCVSVVLASNITVEQRILNLEE

C. elegans (3) --------------------------------------------------

WINLQVNNTIEQILDFTEDSDKLLGFPTRVHVTTNSTLTSDH--PLFITA

-MLVQTPVTTEQFTSIS-----------RTLCPHNNMFDEDA--------

NYPQIINNTMSYLFLY-DYLPNSTFEPPRVKVTLIEPEDNSIVDPLIVVV

NYPQIINNTMSYLFLF-DYLPNSTFEPPRVKVTLIEPEDNSIVDPLIVVV

NYRTVINKNVSYLFLY-DYMLNSTLEPPRVKVTLVEPENHDIIDPLIVVV

---MEINKTVSYLFEYPNYKDVSDLEPPRVTISSTSADES---YPIIIVV

KLYNFQANNATVPVLVFNKTENSTIYPARITTSSDAATAEN---PLLFAA

AYQFAININVEYVTSM---ETVRIEVESNATSN----------LPLIVVV

FYTFSINKSVEYILEF---SAPELKYPPRVTINSSDAQIK---TPLMVVA

VMPFLFNQTTEHVLVF---PTSDSIYPYRVKAWSSGAKLAS---PVLVVV

VMDFTLNSNIKYLLLY---HPQNNNNPYSIKAWSDSASPQN---PILIVV

EYTLVVTPSIEFILQFVP-NEDQAEFPSRLWVRSVGGDTSR---PLLLTA

--------------------------------------------------

TQQKGVSSWELPLVLQTDDY--FLMLNDMGRTLCPHDAGSDIRR------

-------------------------------------EACDAPS------

RHRSGVISWQLPYIE---KQ-QEIKYYKAAHILCPLLSASNN--------

RHRSGVISWQLPYIE---KQ-QEIKYYKAAHILCPLLSASNN--------

RHRSGVISWQLPFIE---KQ-QEIKYYKAAHILCPSLSASNN--------

QQRRSVLSWKLPLTIQ-SKQ-NSYAYHRTSRTLCTDLEDVQNPE------

WQKSQLTTWEVPRLVP--SN-KNLLYRNVTRTLCPHYVNINSTA------

RQKKEFLSWQIPLIVKSMYF-NNSEYNKTSRTLCSTNYNHNGLK------

RQPKELLSWQLPMVLESDTG-NHN-FTKISRTLCHDMYRDYASRG-----

RQEREVISWQVPFVVDTTMKDGVVHFHNTSRTLCHNDMPRIAKAKATSRI

NQGIDTLSWSVPYSIFSQSE---VYYH-TSRTLCDSHN------------

RTKTGATTWQLPYQSG------SMLMSELERTLCWDGSPTDAVGAPSECE

--------------------------------------------------

-----ESPPTVQLTTSSSANVSVDIKLKRVEDFYIELGKVNEVIVNPSSP

-----LNTPIVHLTSSSSEQLKVTILVEKVQDFYIKINQTINITVSPSQP

-----ESRIVVSVSTNSINNITFILRLDIQKSFNVLLNQEVSFNLSPSEP

-----ESRIVVSVSTNSLSNITFILRLDIQKSFNVLLNQEVSFNLSPSEP

-----ESGIVVSVSTNSPMNITFILRLDVQKSFNVLLNQEVSFSLSPSEP

----GEHEMVISVSTSSPSNVNFSLEVVKQQNFSIKLEDEYETVITPSEP

----HMDSFFITVSIDA-SNTDFTIRAQLVDDFVVDVGKPISFTVSPAHS

---QEKEFMIISVSTTNHQNISFILNVTKEHNFYLSTGENKTVEISPSQP

---ITVDSPIVSVSTAAPRNVTFTVQVDYQKDFFIKPSVKYNFNITPSEP

LPIQLSQNFIIALSTSSLANVDISVMVEEERDFYLQEGRPYEVSVSPSES

------QNFTITLSTSAPTNTKLSMIVEEERFFHLVNGKRHTIEISPSEP

G-AGSQRGFTLHLASACAAPLTVTLRAAPARDWLLGFQARTTVTATQTGP

----------MRFTSLSIAFLACALVVSGS-----AIREKRQCGCAQPQQ

RYYYFSFDQNPWN-------------VSHAAGGPLDGTQRYN------YN

KYYFYPFKKGSGKTVDFDEQKMRKEYICGASGGRGHGEVQRNEVGYGWLS

VYYYYSFKQNSSMV------------------------------------

VYYYYSFKQNSSMV------------------------------------

VYYYYSFKQNASMV------------------------------------

AFFLYNFSENVSSV------------------------------------

QFYSFTFPDYLSSG------------------------------------

IYYGYTFSGQVESS------------------------------------

RFYFYNFTANITESPN---------------------------------S

KYYYYKFHDKKNTS------------------------------------

RYFSYDYVPQSHSSL-----------------------------------

AVNYYDFIPGQNSVR-----------------------------------

SQCSCQQVQQTQSC------------------------------------

IPKSVILVIESDDEICATVSIQNNSCPVFDNEREVKYKGYHLTMSSQGGI

RPENVIVMIESDDELCAVVSIQNFSCPVFDNERDILYDGYYLTMTRRGGI

-----LLHVKSDDSICMTLSIQNSSCPVFDSLETVQYDGLRQTVSKTGGI

-----LLHVKSDDSICMTLSIQNSSCPVFDSLETVQYDGLRQTVSKTGGI

-----LLHVISDDVMCMTLSIQNSSCPVFDSLETVQYDGLRQTVSKTGGI

-----LLEIDSPDKTCMTLSIQNISCPVFDLEHTIQYRGDWETVSSKGGI

-----VLRIKSDNSVCMSLSVQNFSCPVYDQENNLKFTGYWETITKSGGM

---SVIVHVKSDSDICMTVSIQNISCPVFDLERNIEFSGYWQTVIRQGGI

NYETVILEVFSDDFVCMTVSIQNASCLVFDTNQDITFRGFYETVNTQGGI

----AMIEINSDDDVCLTVSIQDSFCPVFDLDKDITYEGKYQTINRKGGM

----VTIEIDSDDETCLMVSVQKHTCPVLDLNNFINYQGFHQTILTKGGM

------LIVESEDEVCATISVQRYTCPLAETIEDIDLTTLRMTVMRSGAV

----------SCQSAPVQQQSPSCSCAQPQQTQQVQVQSTQCAPACQQSC

TLTQAMFPSGFYVVLIVRQSDADCTGASET-------------EDAPKSF

TLTQDTFPIGFYIVFIVKTSDEDCKEPGTNGSVPAVARLLGWGDNIQVSS

IISKDEYPLGLFIVFVVHSDDSACH----QGNYQ---------------A

IISKDEYPLGLFIVFVVHSDDSACH----QGNYQ---------------A

IISKDEYPLGLFIVFVVHSDNSACN----RGNYQ---------------A

MLTRQDFPEGLYIVFDLHSDDSDCI----SSTVDGP---------EVDVS

LLTKEAFPLRVFLVFVVHSNDGDCSGHI-EGNSG---------------S

TVPKEEFPLGFFVVLVVKSDDTDCY-----GTPT-------------MIP

TIPKYKFPYGFFAVFVAKPDDSDCT-----GIPS-------------LYY

TIRQREFPDGFFLVFVAKADNYQCSQKHSVLLVEHR---------KQHLI

RIRKKYYTGGFFLVFTVVED--EVCKKKDLPIIPN---------------

QLSRSLYPMGFYVVSLVRPDDAACSGEPAPEDDWLLEAALWAHTDRPSPP

QQQCQASPS---VSQCQPQCQQQCQAQCTPMYNP---------------P

Trans-membrane helix-1

PAKRSKTFRLKIIATISYQEYLVGA----LVSAALVLLVALFVLAL----

TEGRVKNFRFKIVETISYREYLIAAGATVLFYASFYLAFFVFVLYQ----

INFRTKSINFVVKPTVDFN-YQIINCLIVIVIFIFILV--FTTFFY----

INSRTKSINFVVKPTVDFN-YQIINCLIVIVIFIFILV--FTTFFY----

TNSRTKSISFVVKPTVDFN-YQIINCLIVTFIFISILV--LTTFFY----

EADRKKVIRFKLNKNLNYE-DYLFASLAVLLSFSSVFI--LSGLLL----

PADRIKRVELEIIPSITYQ-DYLIAMAAVLIAFALMYIGAFGVFVW----

SRN--KKVILTINASITKK-DYIIASGIVVCVIFSFCITYVVSTVI----

DTNRTKTITLIVKPSISYQ-DYVNAVIATLSSIGIFYFVLIAGFIF----

LANRTSTITFTINKGINGK-EYEIASLATLGALLSFCIVSTIMIFA----

-QNQSSTVHFTVTENIESKNHYIPAVFIVLACFILFSFVAIAIFCV----

ATLRQKTFTLTVRASLSRAQYMVGAGVTVAVFLLFYAGFAALVLAQRWPA

TTTTTTQAPVVQYQ------------------------------------

--------------------------------------------------

--------------------------------------------------

--------------------------------------------------

--------------------------------------------------

--------------------------------------------------

--------------------------------------------------

--------------------------------------------------

--------------------------------------------------

--------------------------------------------------

--------------------------------------------------

--------------------------------------------------

CARLTAPRAVLADAHKSESGALSEGVSVTGVTAETGVTSVTGVTSVTGVT

--------------------------------------------------

------------------------------------LLPCPCRCTEEVTV

------------------------------------SRKSTGVEHQDQET

------------------------------------HTKGDDLKTIDIIQ

------------------------------------HTKGDDLKTIDIIQ

------------------------------------HAKRDNLKTIDITQ

------------------------------------CCTRKSPETVTVS-

------------------------------------AHKRKERNELSEEY

------------------------------------SKVKRNRQMKEEIL

------------------------------------CSKRGYVPRQMEYV

------------------------------------FTRWGTISKFRPSG

------------------------------------FERYRKK-KIAKNT

SDAGTPVRTARRRRGSDATFDSSDASDTDSEEESPAVTNDTITNNMIANP

--------------------------------------------------

VVEESS----------PSTSR-EDSAETDTQP--------ILEAGAADES

IVRSSHGSEGQSSGEGPSLPRPTDGSSVDSQSDTESEYSTLDDVTTDKEL

EEPSTSICSTPVNIQPDNVS--------YDSSLDETDIDILKSPEPWKDL

EEPSTSICSTPVNIQPDNVN--------YDSSLDETDIDILKSPEPWKDL

EEPSTSICSTPVNIQPDNVS--------YDSSLDETDIDILKSPEPWKDL

EESCTVISAGEIINPNDSVS--------ADSSLDEEETD--KSSKESKEL

DSFCGEASVVTQTLSGADEV--------GFFNSDQLSSNTSTLARPRERI

NQESEHINEPIPSPSTVEESG--PVSIDDDSSLDEDDIDIMEDALSDKEI

SSEP-----ATPSTCLGEEV--------DEISLDETEYDVVSEADQDKSI

DELDADWEEPPEPP----------------------------ITRELKHE

EQIAMNVDEKTEEE----------------------------IHEE-RDE

TASSSAANPTTSPGTPGNHGAASPPDRANGAVTEGDAIERSTVQEETSRP

------------------------------------------QCQPVCQQ

Trans-membrane helix-2

WSREHALTVGKLTRAPPDTLARRSDRYFWGALTLAVVYALPVVQLLLTYQ

YRYGTKLCLADLSRCRARVLATRSNRYLWTVLTVSVFYTLPVLQLVVTYQ

IRTKACLYVSDLSKKDHRILKAKSRLYVWNLITVAVFYSLPVTQLVFTSQ

IRTKACLYVSDLSKKDHRILKAKSRLYVWNLITVAVFYSLPVTQLVFTSQ

IRTKACLYVSDLSKKDHRILKAKSSLYVWNLITVAVFYSLPVIQLVFTSQ

VGTKTFLFVSDLSKKNPQAVQAKARLYFWNLLTVAVFYSLPVVQLVFTYQ

IK----LCLNDLAKVNHDVMRRRSNLYLWNLMTIAIFYSLPVVQLVLTYQ

IRTKLVLSVCDLARKDPKILRHKSRLYLYYLITVAIFYTLPAVQLLITYQ

RLGKSVVYLSDLARKDPRVHKYKSYLYLYNVLTVALFYGLPVIQLVVTYQ

LLSRQALTVNLLARAPEKDKR-RSYNYLWHILSIAIFYSIPVVQLVITYQ

NNQQIPNNVADFSQNTQKNQK-RSMNYLWQILNVGLFYIIPVIQLVVTLQ

FGLPARLHVAALARRGRRVLRARSDRYLHTLYTVAVFYALPVLQFVAAFQ

QCQSTCVQQQQPAAQCQPQCQQQCNVACDSPSTTTQAPQVIQIQLEIQQA

Trans-membrane helix-3

RMVFQTGDQDLCYYNFLCAHPLGT----LSDFNHVFSNVGYVLLGAVFAG

RLLNQSGNQDLCYFNFFCAHPLMM----LSDFNHVFSNLGYVVLGALFLL

KMLIETGNQDLCYYNFLCSHSFILGPWKFSDFNHIFSNIGYIFFGLLFIL

KMLIETGNQDLCYYNFLCSHSFILGPWKFSDFNHIFSNIGYIFFGLLFIL

KMLIETGNQDLCYYNFLCSHSFVLGPWKFSDFNHIFSNIGYIFFGLLFIL

KVLNMSGNQDMCYYNFLCSHRLLQ----ISDFNHVFTNIGYILLGLLFIL

KVLNSTGNQDLCYYNFLCSRKLGQ----FSDFNHIYSNIGYILLGILFLL

HVLHVTGNQDMCYYNFLCAHPFQA----LSDFNHVFSNIGYIMLGFLFIF

RALNETGQQDLCYYNFLCAHPLGV----ISDFNHVFSNSGYVLLGLLFLG

RVVNRTGDQDMCYYNFLCANPAFG----LSDFNHIFSNVGYIIVGILFLG

SFLIQTGDFDLCYYNFRCANPLWI----ISDFNHVFSNIGYILMGIVFSI

VMLNISGSLDMCYYNFLCAHPAGG----LSDFNHVFSNLGYLLLGALFML

QAQCQPQCQQQCQSSCVQQQQQ--------------SNQCEPACNTQCSD

Trans-membrane helix-4

QVRFRQVKSRQRPE---NLGIPQHYGLLYSMGLALSMEGLLSACYHLCPN

QVWRRQRIMRNEPEEKKQKGIPQHFGLLYAMGVALISEGFLSAAYHVCPN

ITYKRECVNIP----NKKFGIPNHYGLYYAMGSALAMEGLMSACYHVCPN

ITYKRECVNIP----NKKFGIPNHYGLYYAMGSALAMEGLMSACYHVCPN

ITYKRECVNIP----NQKFGIPNHCGLYYAMGSALAMEGLMSACYHVCPN

LVYRKDAACQL----HSTKGIPHHFGLYYSMGTALMMEGILSACYHICPN

IVYRRHATEK-----KKATGIPQHYGLYYTLGAALFMEGILSGCYHFCPN

LTSFREHNEFDKEK-NKCYGIPQHYGLFYAMGTALIMEGILSGSYHVCPN

ITYRREITHKDLNF-ERQYGIPQHYGMFYAMGVALIMEGVLSGSYHVCPN

VVLHRQTKIPN-----SSTGIPVHYGVYYAMGIALIIEGILSACYHICPS

NVFYRHFYSPP-----LTTGVPANYGVFYAMGAALIMEGVLSGCYHLCPN

QLQRRKRNRKRAPR-HEEYGIPAHYGLLSSLGAAMMVVALLSASYHVCPN

ICQQTAQATQQ----------------VYNQNMNQNTNTQMYNPYNTNTN

Trans-membrane helix-5 Trans-membrane helix-6

KMNFQFDSSFMYVIAVLVTLKLYQNRHSDIIPSAHSTFMILAVIMTIGLF

SMNFQFDTSFMYVTSALCMVKIYQSRHPDINARAHATFGVLALIIFIGLV

HSNFQFDTSFMYVICMLSMIKIYQTRHPDINANAYLVFGVLALVIILGLT

HSNFQFDTSFMYVICMLSMIKIYQTRHPDINANAYLVFGVLALVIILGLT

HSNFQFDTSFMYIICMLSMIKIYQTRHPDINANAYLVFGVLAFVIILGLT

HSNIQFDTSFMYIIAMLSMLKIYQNRHSDINASAYTTYLVLACVIFIGMC

HSNFQFDTSFMYILAIISILKIYHSRHPDINASAYTAFGLLAVVITLGMC

RSNFQFDSSFMYIITVLCMIKIYQTRHPDINARASVTFAMLAFIIFINLM

TANFQFDSSFMYVMAVLCMVKLYQNRHPDINATAYATFGVLAVAILLGMI

QSNYQFDTSFMYVMAVLCMIKLYQNRHPDVNATAYATFTVLGMAIFLAMI

ETNFQFDTSFMYVMIVLCLVKLYQNRHPDVTPTAYTTFSILGATILCGTI

SLNFQFDTAFMYVLAVLCMVKIYQSRHPDINARAHATFGVLAVFIALVVW

-QNANCAPACQPACDNSCTSQQTQPMYQPYDTTTEAPAQVIQIVLQTSVA

Trans-membrane helix-7

GILHPSAGFAASFTLL-HLGACLVLTLKIYYAGRFKMDRRVLLR----AY

GVLNANVYFWVAFTAL-HLLTCFFITFQIYYLGRFKLDMGWVRA----AS

GIMYEGPILFVLFTCL-HLIMIFWLSAQIYYMGRWKLDKKTPKR----FL

GIMYEGPILFVLFTCL-HLIMIFWLSAQIYYMGRWKLDKKTPKR----FL

GIMYEGPILFILFTCL-HLIMIFWLSAQIYYMGRWKLDKKTPKR----FL

GILNGSFIFYVVFTAL-HIITCFFLSLQIYYMGRWKLGFHSFKRTIIEFF

GVLYISTGFYVAFCIF-HVCICLFLSAQIYYMGQWNLDSSHSQNIKKIII

GVLNGSIYFWILFTIT-HLLTCLFMTIQIYYMGRWKFRA-LLTRVLQNCK

GILEGNLYFWIVFTII-YLLSCFYLSIQIYYMGCWKLDAGLAMRVWRICV

GILNGSLTVWIVFVVI-YSLLCAYISFKIYFIS------FVFDGFKQLKQ

GIVFKAPPVFIVFVTIAYLVLLIYASLNIYHFG------TARNFLR--RC

GVLGGGPLFWSVFTVL-HVFTFLLLSLRIYYVGQFRLE----------KS

QSSQCAPQCEQSCQQQ----------------------------------

Trans-membrane helix-8

AHVAARGWRSLLPAHPYRAGLLGLANLANWSLAGYSVYSHHN---TDLAR

RGAALR---------PSRGLMLLLANLINWGLAGYGVAQHS----RDFAS

NHIMTAP-NPCRPKYPNRMVLLSFGILINLGLAVSHWIIKF----GNFGN

NHIMTAP-NPCRPKYPNRMVLLSFGILINLGLAVSHWMIKF----GNFGN

NHLMTAP-NPCKPKYPNRMVLLSIGILINLGLAISHWIIKF----GNFGN

TNLRAGL-RHCKPMYPNRMVLLILGNACNWGLAVHLWMSNR----SNFAT

SKIRSGRENYCRPEYPSRMALLVLGNVCNWALIASGLFFHM----GDFAT

HDARSGIRYLFRPLYIGRFFMLVIANLWNIALAVIGNIYQE----KNFAT

YEFWSGPLNVIKPIHKARMCLLIIANLCNWGMAFWGVYKHQ----KDFAL

SLKSSNKVEAIAPIRKSRFALLVIANIINYAMLITGLCLYN-TGVTDFGT

CLRNSEVPRPIQSPNTHRWWLLLLAITVNILLYGLGLILFYHTKTIDFAT

SLAVAARGLRARPLYTPRLVMLLIANAANWGFAIYGLLTHAG----DIAT

-------------------CVQQQQPAAQCQTACQSSCSNS---------

Trans-membrane helix-9 Trans-memb-

QLLAILMGNAILYTMFYMVMKLVN--------RERILARTWMYCILAHVA

HLLLVLMTNLFLYTLFYIVMKLLH--------RETITCYTWVFIVLTYSS

YLLILFMVNLILYLSFYIVMKLIS--------KEKLHFWPLLYILLAVIF

YLLILFMVNLILYLSFHIVMKLIS--------KEKLHFWPLLYILLAMIF

YLLILFMVNLILYLSFYIVMKLIS--------KEKLHFWPLLYILLAMIF

YLLIIFMANLILYLTFYITMKLLS--------GERILLQPFLYIIFAVIF

YLLAILLCNLLIYFSFYIIMKLIL--------GEKILFQPLIYTVLALIS

FLLAILMSNLILYTTFYIIMKICH--------KERILLQPCIYIVLSIVF

FLLAIFMGNTLLYFSFYIVMKIIN--------KERVNKLSLFFLSLSVLC

FLLGLLMGNSVLYAVFYTGMKLVN--------GERICFEAIIYGLLAIAA

FILQILAGNAFLYTVVYTCMKIKCTSVRECTCSEKICAQAIIYGFLALVT

HLLNVLLCNTLLYIVFYVLMKLLH--------GERIRWYSWCFLAAAAAC

--------------------------------------------------

-rane helix-10 Trans-memb-

WFLALRLFLDSKTKWSETPAQSRQHNAPCSSLSFYDTHDLWHGVSAAALF

WAGSSYFYLDQNTNWALSPAQSRERNAACSVLRLFDAHDAWHAMSAVAMF

WSASLYFYVHKSSSWTLSAAESRTYNTPCTFMDFYDNHDIWHFLSAISLF

WSASLYFYVHKSSSWTLSAAESRTYNTPCTFMDFYDNHDIWHFLSAISLF

WSASLYFYMHKSSSWTLSAAQSRTYNTPCTFMDFYDNHDIWHFLSAISLY

WGASGYFFMSRSTNWQLTPAESRTYNKSCMLLKFYDNHDIWHLISAGSMF

WAAAGYFFYHKTISWKLTPAQSRTFNRPCVLFDFYDNHDIWHFLSAVSMF

WAAALYFFINKTISWELTSAQSRHYNKPCELLHFFDSHDIWHFLSALAMF

AISAMYFFLNKSISWSRTPAQSRQFNQECKLLRFYDFHDIWHFLSAIGMF

WATAAVYFLDNATLWTVTPAESRQWNQECIVMSFYDKHDVWHLLSAPALY

WVLAGVFFFTEASKWTESPAQSRQLNKQCIFADFYDSRDLWHFFSSLALY

WVPALYFFTSGSTDWSATPARSRHRNHECRVLQFYDSHDLWHMLSAAALY

---------CQAAQPATTACQQSPQQSSCSCQANYSPCGNGQCCRRK---

rane helix-11

LSFNMLLTMDDALRDTPRDQIPTF---

LSFNMYLTLDDGLAGADRARVPVF---

LSFMVLFTLDDDVNSKPTATIPVFA--

LSFMVLFTLDDDVNSKPTATIPVF---

LSFMVLFTLDDDVINKPTATIPVF---

FSFMVLLTLDDDLKDKERKLIPVF---

FSFMVLLTLDDDIANVDRSLIPVF---

FSFMVLLTLDDDLIDVHRSQIPVF---

FTFMVLLTLDDDLSHTHRNKIVVF---

LTFMFLLSLDDDLVDIKREEITVF---

FTFMYLLCIDDNLYTN-RADIPLF---

FTFNVMLTWDDGLSAVKRTEIAVFELI

---------------------------
