# Supplementary material for: siRNA Machinery in Whitefly (Bemisia tabaci)
Source: PLoS One. 2013 Dec 31;8(12):e83692. doi: 10.1371/journal.pone.0083692 (PMC3877088; doi:10.1371/journal.pone.0083692)
Supplement: Table S1 — Sequences used for various analyses during study. (DOCX) [file pone.0083692.s006.docx]

| Dicer | Insect | Accession Number |
| --- | --- | --- |
|  | *Bemisia tabaci* |  |
|  | *Aphis glycines* | AFZ74931.1 |
|  | *Acyrthosiphon pisum* | XP_003240110.1 |
|  | *Bombyx mori* | NP_001180543.1 |
|  | *Caenorhabditis elegans* | NP_498761.2 |
|  | *Drosophila melanogaster* | NP_523778.2 |
|  | *Tribolium castaneum* | NP_001107840.1 |
|  | *Blattella germanica* | CCF23094.1 |
|  | *Laodelphax striatella* | AGE12616.1 |
|  | *Nilaparvata lugens* | AFK73581.1 |
|  | *Danaus plexippus* | EHJ65725.1 |
| R2D2 | *Bemisia tabaci* |  |
|  | *Aphis glycines* | AFZ74932.1 |
|  | *Acyrthosiphon pisum* | NP_001155644.1 |
|  | *Apis mellifera* | XP_001121349.2 |
|  | *Bombyx mori* | NP_001182007.1 |
|  | *Caenorhabditis elegans* | NP_499265.1 |
|  | *Tribolium castaneum* | NP_001128425.1 |
|  | *Drosophila melanogaster* | NP_609152.1 |
| Argonaute2 | *Bemisia tabaci* |  |
|  | *Nilaparvata lugens* | AGH30327.1 |
|  | *Locusta migratoria* | AGO85972.1 |
|  | *Tribolium castaneum* | EFA04626.1 |
|  | *Aphis glycines* | AFZ74933.1 |
|  | *Acyrthosiphon pisum* | XP_001944852.2 |
|  | *Apis mellifera* | XP_395048.4 |
|  | *Bombyx mori* | NP_001036995.2 |
|  | *Caenorhabditis elegans* | NP_871992.1 |
|  | *Drosophila melanogaster* | NP_648775.1 |
|  | *Tribolium castaneum* | NP_001107842.1 |
| Sid1 | *Bemisia tabaci* |  |
|  | *Aphis glycines* | AFZ74934.1 |
|  | *Aphis gossypii* | BP98803.1 |
|  | *Acyrthosiphon pisum* | XP_001951907.1 |
|  | *Apis mellifera* | XP_395167.4 |
|  | *Bombyx mori* sid-1 a | NP_001106735 |
|  | *Bombyx mori* sid-1 b (2) | BAF95807.1.1 |
|  | *Bombyx mori* sid-1 c (3) | NP_001106736.1 |
|  | *Caenorhabditis elegans* | NP_504382.1 |
|  | *Triboliumcastaneum* Sid-1 a | NP_001099012.1 |
|  | *Triboliumcastaneum* Sid-1 b (2) | NP_001103253.1 |
|  | *Tribolium castaneum* Sid -1 c (3) | NP_ 001099128.1 |
|  | *Nilaparvata lugens* | ADI88514.1 |

**Supplementary Table 1**. Sequences used for various analyses during study.
